# Design, Molecular Modeling and Synthesis of Metal-Free Sensitizers of Thieno Pyridine Dyes as Light-Harvesting Materials with Efficiency Improvement Using Plasmonic Nanoparticles

**DOI:** 10.3390/molecules25081813

**Published:** 2020-04-15

**Authors:** Mohamed E. Khalifa, Abdulraheem S. A. Almalki, Amar Merazga, Gaber A. M. Mersal

**Affiliations:** 1 Department of Chemistry, Faculty of Science, Taif University, Taif 21974, Saudi Arabia; 2 Department of Physics, Faculty of Science, Taif University, Taif 21974, Saudi Arabia; 3 Department of Chemistry, Faculty of Science, South Valley University, Qena 83523, Egypt

**Keywords:** tetrahydrobenzothiophenes, thienopyridine, HOMO-LUMO, organic sensitizers, dye-sensitized solar cells, metal nanoparticle, plasmonic

## Abstract

Considering the thiophene unit as an electron-rich heterocycle, it is investigated with the aim of elucidating its potential efficiency for solar cell application. With the introduction of active substituents such as COOEt, CONH_2_ and CN into the thiophene segment, three novel thieno pyridine sensitizers (**6a**–**c**), based on donor-acceptor D-π-A construction, are designed and synthesized. The effect of the anchoring groups is investigated based on their molecular orbital’s (MO’s) energy gap (E_g_). The electrostatic interaction between the synthesized dyes and metal nanoparticles, namely gold, silver and ruthenium, is believed to improve their performance as organic sensitizers. The dye-sensitized solar cells (DSSCs) are manufactured using the novel diazenyl pyridothiophene dyes, along with their metal nanoparticles conjugates as sensitizers, and were examined for efficiency improvement. Accordingly, using this modification, the photovoltaic performance was significantly improved. The promising results of conjugate (**6b**/AgNPs), compared with reported organic and natural sensitizers (*J*_SC_ (1.136 × 10^−1^ mA/cm^2^), *V*_OC_ (0.436 V), FF (0.57) and η (2.82 × 10^−2^%)), are attributed to the good interaction between the amide, methyl, amino and cyano groups attached to the thiophene pyridyl scaffolds and the surface of TiO_2_ porous film. Implementation of a molecular modeling study is performed to predict the ability of the thiophene moiety to be used in solar cell applications.

## 1. Introduction

Modern organic solar cells (OSC) are considered as third generation solar cells, as they were first fabricated by Grätzel et al. in 1991 [1]. They have remarkable advantages among potential renewable energy sources such as lower cost, simple fabrication and lower toxicity, and they are environmentally eco-friendly [2]. Dye-sensitized solar cells (DSSCs) of an organic nature are superior to metal-based sensitizers regarding the cost, environmental issues and higher molar extinction coefficients [3]. Typically, the common architect system (D-π-A) for DSSCs consists of a hydrophobic electron-rich donor π-conjugated bridge unit and a hydrophilic electron-deficient acceptor [4]. In this structure, the highest occupied molecular orbitals (HOMO) and the lowest unoccupied molecular orbitals (LUMO) are mainly localized around the donor and acceptor parts of the dye molecule, respectively [5]. This orientation favors the electron injection and slows down recombination between electrons in the TiO_2_ photoanode conduction band (i.e., the semiconductor and oxidized molecules) through the anchoring/acceptor group adsorbed on its surface (i.e., a cyano, carboxylic or phosphonic group) [6,7]. The sensitizer is illuminated by the light; thus, the excited electrons will transfer from donor to acceptor terminals through the π-conjugation bridge and then inject into the conduction band. The design and mechanism of organic sensitizers for DSSCs have been explained in detail by Obotowo et al. [8]. The efficiency of a DSSC as light harvesting matter depends mainly on the nature and the amount of the adsorbed dye molecules on the photoanode [9,10] and the thickness of the photoanode as well [11]. To achieve the most optimized D-π-A dyes, they should be designed for broader absorption in the near IR region, minimizing the energy level mismatch between the oxidation potential of the dye’s excited state and TiO_2_ conduction band, and the HOMO and electrolyte as well, reducing the rate of charge recombination at the photoanode-electrolyte interface, prohibiting dye aggregation and increasing the stability of the DSSC [6]. Thiophene derivatives (e.g., bithiophenes [5], oligo thiophenes [5], aryl thiophenes [12] and aryl bithiophenes [13]) have attracted the attention of many researchers to synthesize donor-acceptor substituted π-conjugated systems, such as formyl π-conjugated systems, through cross-coupling/metalation followed by DMF quenching [5] and Vilsmeier-Haack-Arnold formylation reactions [13,14]. Plasmon resonance employing noble metals (i.e., Ag and Au) has been recently introduced to the DSSCs, where the metal nanoparticles’ surface plasmon resonance phenomena are assumed to enhance their light harvesting efficiency [15]. It was reported that silver nanoparticles achieved light trapping in solar cells at a higher rate than that of other noble metals in visible range, due to their higher relative scattering efficiency [16]. In the light of the previous literature, it is of interest to design new, organic DSSCs of thienopyridyl thiophene dyes, identify their electrical characteristics and examine their utility in solar cell applications. In addition, the influence of the plasmonic resonance of different metal nanoparticles (namely gold, silver and ruthenium) on the redox properties has been investigated, aiming to enhance their light harvesting efficiency. Computational modeling study has also been performed in order to predict the reactive sites of the dyes’ molecules and their ability to serve in the field of solar cell applications. Density functional theory (DFT) method exhibits outstanding explanation of the structural, spectral and electronic properties of the studied molecules [17,18,19]. The B3LYP calculation functional method has been chosen to predict the energetic, electronic and spectral properties for many molecules in high precision [20,21,22].

## 2. Results and Discussion

### 2.1. Chemistry

As a part of our continuous efforts for the synthesis of novel organic dye-sensetized solar cells [23,24,25,26], the thiophene nucleus has particularly attracted our attention. In this regard, we herein report the synthesis of novel scaffolds of diazenyl pyridothiophene derivatives (**6a**–**c**) as metal-free organic sensitizers. 

**Scheme 1:**
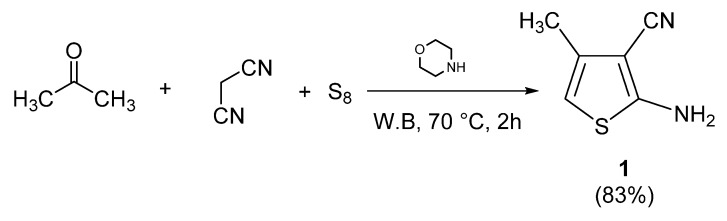
Preparation of 2-amino-4-methylthiophene-3-carbonitrile (**1**).

The synthesis procedures are carried out using the 2nd version of Gewald strategy to achieve the final products starting from preparing the 2-amino-3-cyano-4-methylthiophene compound (**1**) [27]. As shown in Scheme 1, the acetone mixes readily with sulphur, malononitrile and the base catalyst (morpholine) to afford the target 2-aminothiophene derivative (**1**).

**Scheme 2:**
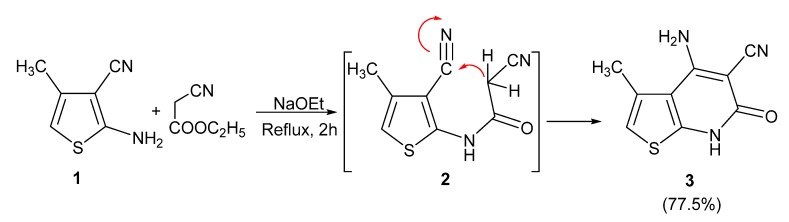
Synthesis of 4-amino-6,7-dihydro-3-methyl-6-oxothieno[2,3-*b*]pyridine-5-carbonitrile (**3**).

The mechanism of this reaction was proposed by Gewald [27]. The structure of the prepared starting compound (**1**) is secured by its micro and spectroscopic analyses and found to be in good agreement with the literature [28]. The cyclization of 2-amino-3-cyano-4-methylthiophene (**1**) with ethyl-cyanoacetate in the presence of sodium ethoxide affords the 4-amino-6,7-dihydro-3-methyl-6-oxothieno[2,3-*b*]pyridine-5-carbonitrile (**3**)**,** as depicted in Scheme 2 [29].

This reaction is assumed to proceed through formation of the non-isolable intermediate (**2**) followed by intramolecular cyclization to afford the cyclic pyridone (**3**) according to the Thorpe-Ziegler reaction mechanism [30].

**Scheme 3:**
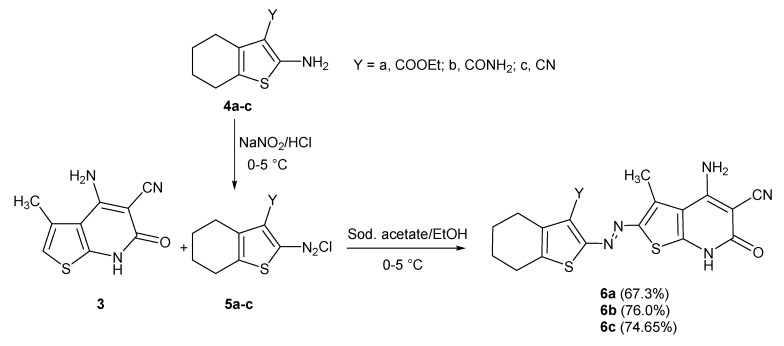
Synthesis of thienopyridyl-azo-tetrahydrothiophene derivatives (**6a**–**c**).

On the other hand, preparation of 2-amino-4,5,6,7-tetrahydrobenzo thiophene derivatives (**4a**–**c**) was very interesting with the aim of coupling with the precursor compound (**3**) to collect the new products of (**6a**–**c**), as described in Scheme 3. Three components’ reactions (cyclohexanone, active nitriles and elemental sulfur in ethanol) in the presence of a catalytic amount of morpholine undergoes Gewald synthesis to give the target products (**4a**–**c**) [31,32,33,34].

Therefore, diazotization of 2-amino-4,5,6,7-tetrahydrobenzo[*b*]thiophenes (**4a**–**c**) in HCl and sodium nitrite gives the corresponding non-isolable diazonium salts (**5a**–**c**), which undergo coupling reactions with 4-amino-6,7-dihydro-3-methyl-6-oxothieno[2,3-*b*]pyridine-5-carbonitrile (**3**) in the presence of sodium acetate and ethanol to furnish the corresponding diazenyl pyridothiophene derivatives (**6a**–**c**) (Scheme 3).

The chemical structures of diazenyl pyridothiophene derivatives (**6a**–**c**) are confirmed by their spectral data. The characteristic IR absorption bands of compounds (**6a**–**c**) prove the presence of NH_2_ groups at 3322–2935 cm^–1^, the cyano groups at 2209–2210 cm^–1^. Furthermore, the strong bands at 1530–1537 cm^–1^ attribute to the NH bending and the informative bands at 1582–1704 cm^–1^ correspond to the carbonyl groups (ester, pyridone and amide). The ^1^H NMR spectra of (**6a**–**c**) exhibit the highly deshielded NH protons at the range of δ = 9.11–10.96 ppm These findings are supported by similar reported compounds and may be attributed to the tautomerization possibility of 2-pyridone isomeric from the 2-hydroxypyridine form [35]. The NH_2_ protons appear in the range of 6.35–6.56 ppm.

### 2.2. Metal Nanoparticles (MNPs) Characterization

The presence of MNPs was previously confirmed and characterized (c.f. supplementary file S2) [36]. The UV-Vis spectra of the surface plasmon bands appear at 537 and 425 nm, confirming the production of AuNPs and AgNPs, respectively, while disappearance of the absorbance band at 318 nm confirms the production of RuNPs. The high resolution transmittance spectroscopy images (HRTEM) estimate that the sizes of the Au, Ag and Ru nanoparticles with an average diameter of 17, 9 and 5.5 nm, respectively. The results are in good agreement with the literature [37,38].

### 2.3. Absorption Spectra Analysis

**Figure 1:**
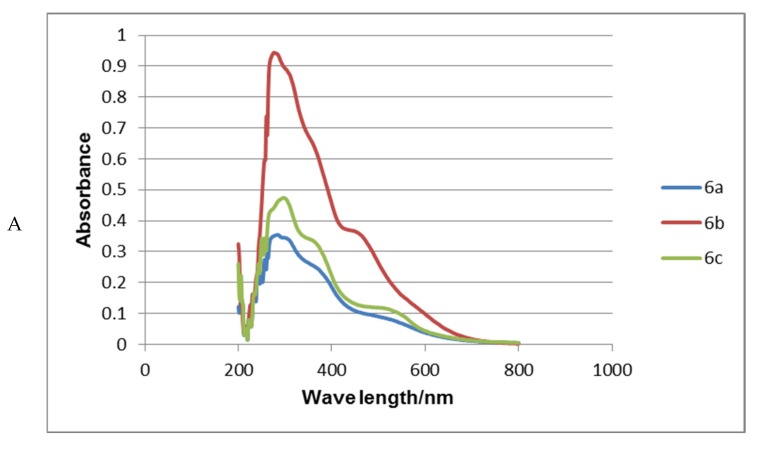

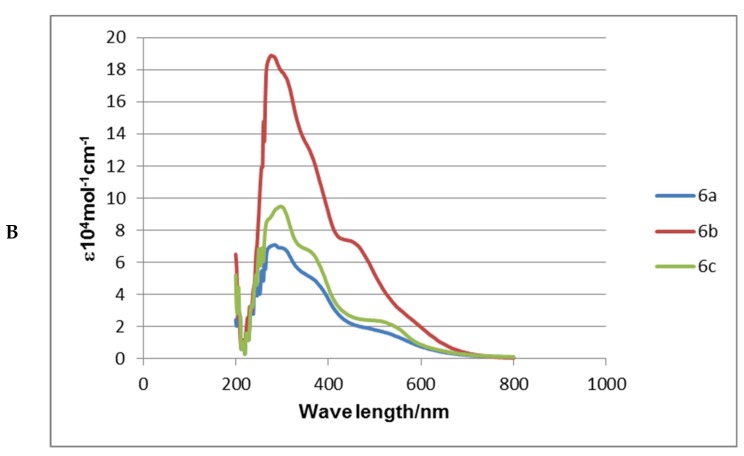
UV-Vis normal absorption spectra (**A**) and molar absorption coefficients (ε) (**B**) of dyes (**6a**–**c**) measured in DMF (5 × 10^−2^ mM).

The UV-Vis absorption spectra (A) of the synthesized dyes (**6a**–**c**) are measured in DMF (Figure 1), where the values of maximum absorbance wavelengths (λ_max_) are affected by the strength of the donor in direct proportion. Dye (**6b**) has a carbonyl group with a stronger accepting character than that of carboxylate and cyano chromophores of (**6a**) and (**6c**), respectively; thus, it shows the highest *λ*_max_. Since the absorption of D-π-A could be shifted towards longer (λ) (i.e., intramolecular donor and acceptor charge transfer), these dyes may be applied for solar cells [24]. Concurrently, the energy levels could be adjusted by incorporating different anchoring nuclei and the π bridge.

**Figure 2:**
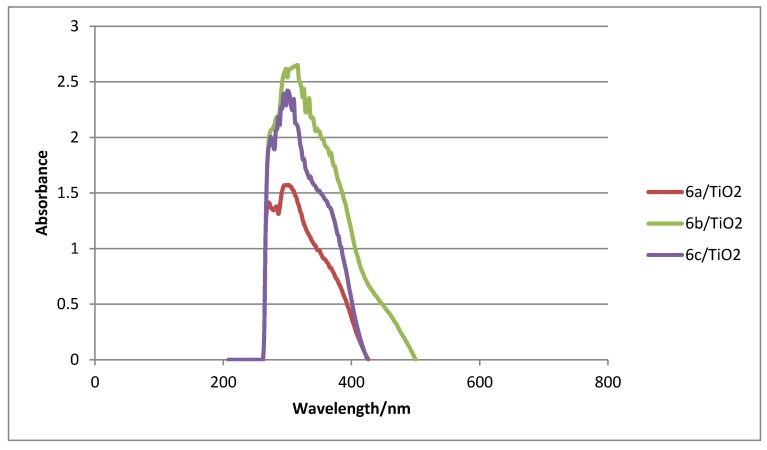
UV-Vis absorption spectra of dyes (**6a**–**c**) adsorbed on nanoporous TiO_2_.

According to the light absorption behavior (Figure 2), dye (**6b**) has the highest adsorption amount on the TiO_2_ nanocrystalline surface and hence is chosen to study its performance in conjugation with different metal nanoparticles.

**Figure 3:**
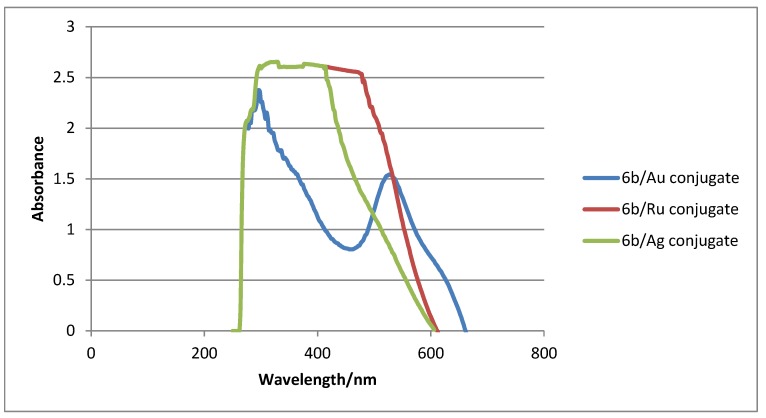
UV-Vis absorption spectra of dye (**6b/NPs**) conjugates adsorbed on nanoporous TiO_2_.

The adsorbed dye/Au, Ru and/or Ag nanoparticle conjugates (**6b**/NPs) on nanoporous TiO_2_ exhibit the role of their plasmonic resonance phenomena in the enhancement of adsorption efficiency (Figure 3). Generally, the conjugation with nanoparticles shows a slightly higher adsorption amount on the TiO_2_ nanocrystalline surface, in the order Ag > Ru > Au, than in the case of dye only.

### 2.4. Electrochemical Characterization

The oxidation and reduction modes corresponding to the removal of electrons from the HOMO and filling the LUMO with electrons, respectively, are determined using cyclic voltammetric measurements [39]. The MO’s energy levels are calculated from the onset oxidation and reduction potentials (E_onset_ for HOMO and LUMO, respectively) of cyclic voltammograms (CVs), where the magnitude of their energy gaps (E_g_) are estimated.

**Figure 4:**
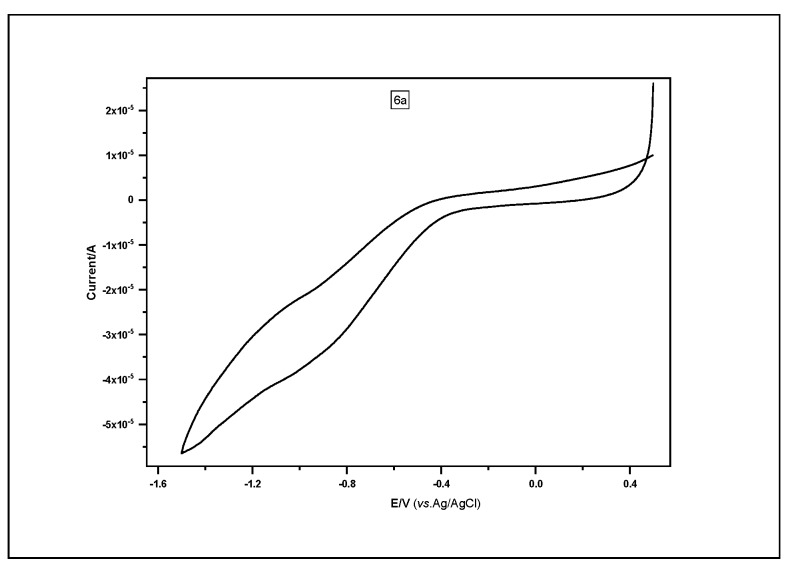
Cyclic voltammogram (CV) of (**6a**) in dichloromethane (DCM) with tetrabutyl ammonium perchlorate (TBAP).

**Figure 5:**
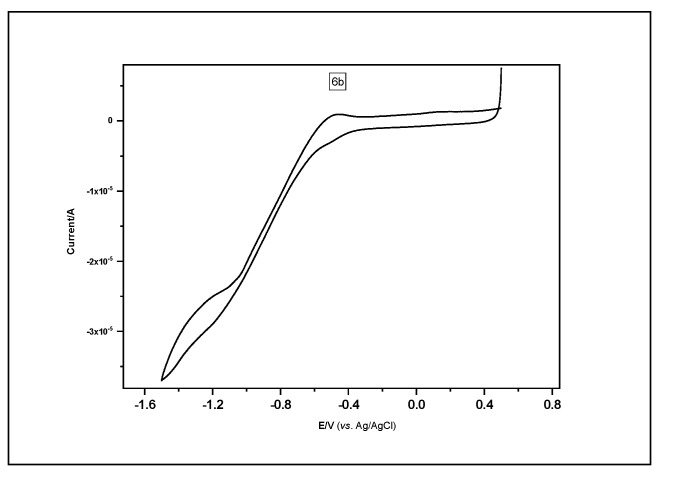
CV of (**6b**) in DCM with TBAP.

**Figure 6:**
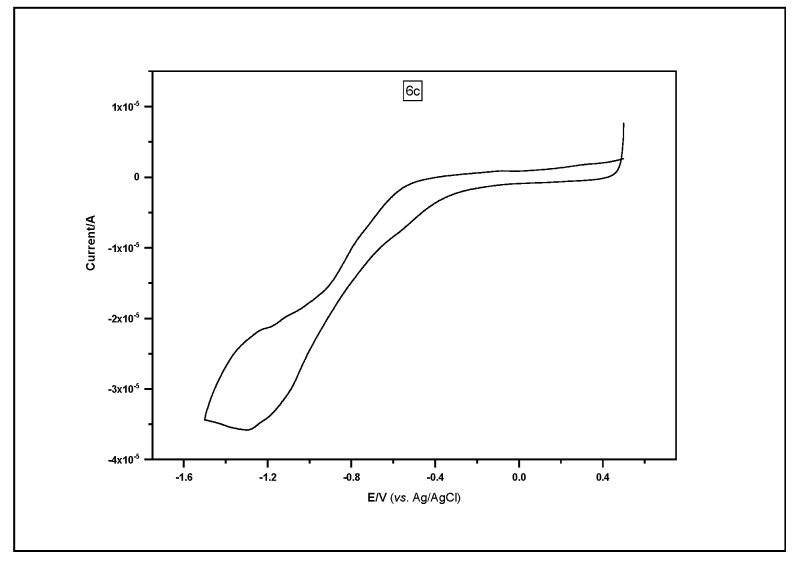
CV of (**6c**) in DCM with TBAP.

Figure 4, Figure 5 and Figure 6 show CVs for dyes (**6a**‒**c**) in dichloromethane (DCM) with 100 mM tetrabutyl ammonium perchlorate (TBAP) as supporting electrolyte, where a single reversible oxidative wave in a positive potential and an irreversible reductive one in a negative potential appear.

From CV analysis, the E_g_ for each compound is calculated using the difference between the electrochemical HOMO and LUMO obtained in terms of the optical energy gap of the absorption edge of the electronic spectra (Equations (1) and (2)).

(1) $$E_{HOMO}=-(E_{\mathrm{onst}\;(\mathrm{oxidation})}+4.4)\mathrm{eV}$$

(2) $$E_{LUMO}=-(E_{\mathrm{onst}\;(\mathrm{reduction})}+4.4)\mathrm{eV}$$

Table 1 obviously shows that dye (**6b**) has the lowest HOMO-LUMO energy band gap (E_g_) among the alternates, indicating the highest performance [40].

Accordingly, dye (**6b**) is therefore selected to study its performance in conjugation with different metal nanoparticles (i.e., Au, Ag and Ru NPs). The E_g_ values of (**6b**/NPs) conjugates are calculated and tabulated in Table 2.

Dye (**6b**/AgNPs) has the lowest E_g_; thus, the conjugation with silver nanoparticles enhances the efficiency of dye (**6b**) compared with ruthenium and gold conjugates in descending order.

### 2.5. Photovoltaic Performance (PV) Study

**Figure 7:**
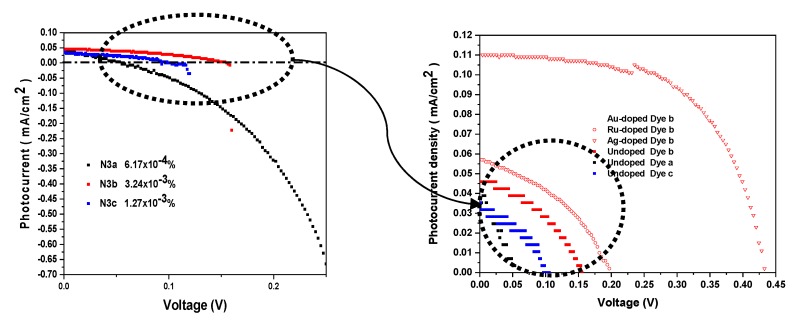
*J*-*V* characteristics of the dye-sensitized solar cells (DSSCs) fabricated from dyes (**6a**–**c**) and (**6b**/NPs) conjugates.

The PV performances of the fabricated DSSC devices using dyes (**6a**‒**c**) along with their NP conjugates on the nanoporous TiO_2_ are studied. The PV parameters and characteristic *J-V* curves of the fabricated devices are revealed in Table 3 and Figure 7.

The PV parameters of dyes are in the order **6b** > **6c** > **6a** under the same experimental conditions. The promising results of the *J*_SC_ values, *V*_OC_ values and *FF* values, compared with reported natural and organic sensitizers, may be due to the better interaction between the functional branches of the thiophene nuclei and the surface of the TiO_2_ porous film [41]. The cell based on dye (**6b**) exhibits the highest efficiency (*J_SC_* = 4.313 × 10^−2^ mA.cm^−2^, *V_OC_* = 0.141 V, *FF* = 0.53, η = 3.24 × 10^−3^%) in accordance with the electrochemical measurements. The finding may be explained by the cell’s higher adsorption on the TiO_2_ nanocrystalline surface among group members, as shown in Figure 2. This phenomenon affords additional absorption sites for the dye, which is correlated with better harvesting efficiency of the photoelectrode, resulting in an improvement in the *J*_SC_. The improvement in the open-circuit voltage (*V_OC_*) for dye (**6b**) may contribute to an increase of the electron lifetime in the DSSC by preventing dark current. The increase of the total power conversion efficiency (η) of dye (**6b**) could be ascribed to its better donating ability.

Furthermore, conjugation of the dye molecules with metal nanoparticles exhibits a remarkable change in photovoltaic performance, as described by Table 3 and Figure 7. It’s obviously clear that conjugation of dye (**6b**) with silver nanoparticles enhances the efficiency (η = 2.82 × 10^−2^%) compared with conjugates with Ru and Au in descending order (**6b**/RuNPs; η = 4.16 × 10^−3^% and **6b**/AuNPs; η = 2.79 × 10^−3^%), which could be explained by its higher adsorption on nanoporous TiO_2_ (Figure 3).

Overall, the reason for the lower efficiencies of organic sensitizers compared with other metal-based sensitizers may be due to the LUMO level being slightly lower than the energy of the conduction band of TiO_2_. This leads to an insufficient electron injection into the TiO_2_ conduction band from the excited form of the sensitizers. Many publications have tried to resolve this issue [42] and this will be considered in our future research by modifying the sensitizers’ design (i.e., photoanode).

### 2.6. Computational Study

Quantum chemical calculations are used mainly for geometrical optimization, predicting the Mulliken atomic charges, calculating the frontier molecular orbitals (FMOs) and determining the shape and energy for the compounds under investigation, with the aid of the Gaussian 03W program suite at semi-empirical PM6 level [43]. The geometrical structure is among the important factors governing the chemical and biological activities [44]. The Mulliken atomic charge is also an important factor that affects reactivity towards an electrophilic or nucleophilic reaction [45]. The FMOs are used to find frontier electron density, where the most reactive sites in the molecule are [46].

#### 2.6.1. Molecular Geometry

**Figure 8:**
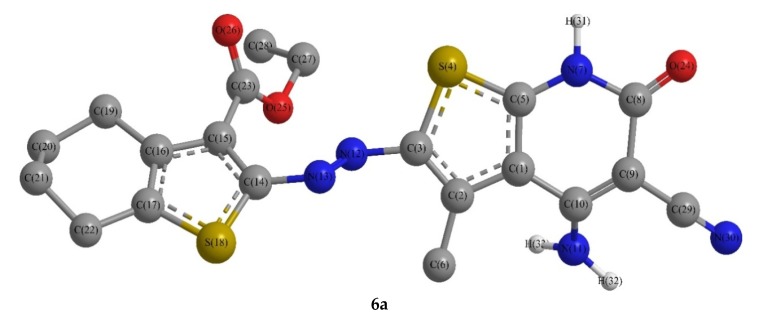

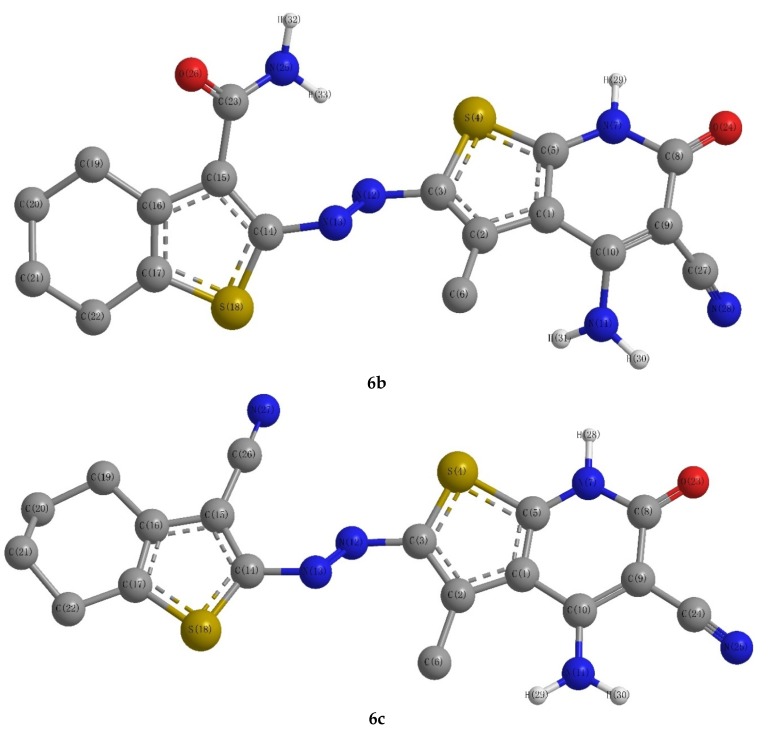
Optimized structures of dyes (**6a**–**c**)**.**

The dihedral angles data of the DFT-optimized structures indicate the twisted geometry of dyes (**6a**–**c**) (Table S1 and Figure 8).

For instance, the thiophene rings are almost planar, whereby the S atoms are lying above or below the plane of other C atoms by ~0.5‒6.0°. The carbon atoms of the thiophene ring fused with pyridine have a distorted planar geometry.

The azo groups are almost planar with the carbon atoms to which they attached, while for the cyclohexyl ring, it is found that both fused moieties are tilted on each other. Moreover, the cyclohexyl rings are bent, and C_thio_-C_cyc_-C_cyc_-C_cyc_ ranged from 42° to 46°.

Concerning the substituents at 3-position on the cyclohexyl ring fused with thiophene, the carbonyl carbon of COOEt and CONH_2_ in the (**6a**) and (**6b**) derivatives, in addition to the cyano group carbon in (**6c**)**,** are almost planar with the thiophene ring. The oxygen atoms of the ester and amide group are found to lie above the thiophene plane by 46.59° and 39.62°, respectively. The cyano and amino substituents on the pyridine ring in the (**6a**) derivative were on opposite sides, while they were almost planar in (**6b**) and (**6c**).

The bond length data, compared with single-crystal X-ray diffraction for peer dyes (Table S2, c.f. supplementary file S1), reveal that the N-N_(azo)_bonds are 1.24–1.25°, which is in the range reported from the X-ray crystals (1.18–1.31 Å) [47]. Moreover, the DFT C_thio_-N_azo_ bonds’ lengths are in the range 1.41 Å to 1.47 Å (X-ray range 1.36–1.50 Å) [48]. Furthermore, the range of thiophene C-S bonds’ lengths are 1.63–1.74 Å for the cyclohexyl fused rings, while those of fused pyridine are in the range of 1.66–1.81 Å, which is in good correlation with the X-ray crystallography data of similar compounds, 1.62–1.76 Å [47].

Finally, the molecular charge distribution is expressed by dipole moment (μ_total_) and is given as a vector in three dimensions, μ_x_, μ_y_, and μ_z_. Therefore, it can depict the charge movement direction across the molecule depending on the centers of positive and negative charges. For instance, the dipole moments components become zero in symmetric geometrics [49]. The investigated compounds have non-zero components in the Z-direction. This confirms their non-planar structure.

As per dipole moment values (Table 4), dye (**6c**) is found to be the most polarized among the group members (μ_total_ = 11.54 Debye), which may be due to the high electron withdrawing character of the cyano group. It has almost a planar structure (μ_z_ = 8.32 Debye). Accordingly, the investigated compounds may be arranged as **6b** < **6a** < **6c** in polarization characters.

#### 2.6.2. Frontier Molecular Orbitals

The energies of the FMOs describe the molecule’s ability to donate and accept an electron. They are calculated and presented in Table 4.

The HOMO-LUMO gap (E_g_ or ΔE) is used for predicting the relationship of the chemical structure and the electronic properties (i.e., frontier electron density and chemical stability) and could explain the ultimate charge transfer across the molecule [50].

**Figure 9:**
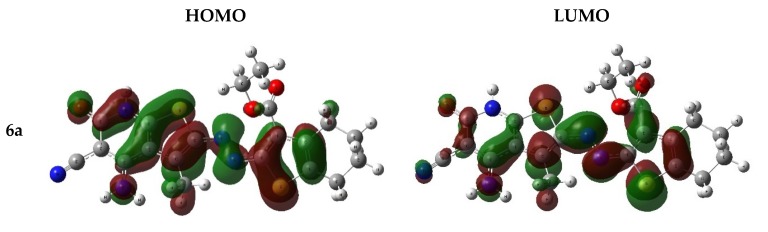

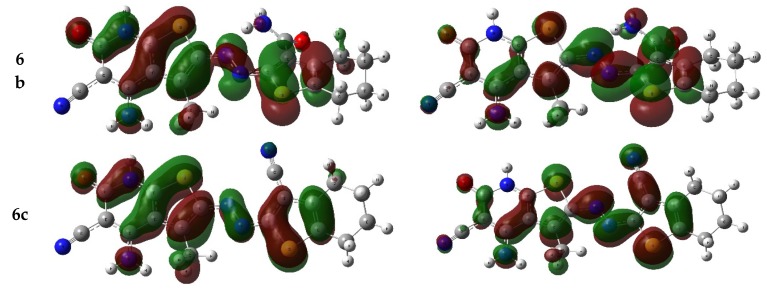
The frontier molecular orbitals (FMOs) of compounds (**6a**–**c**).

The 3D plots of FMOs for the investigated compounds can offer deep perspective about the aromaticity and lone pair (Figure 9) [51]. The positive and negative phases of the wave functions are represented in red and green color, respectively.

The HOMO of the (**6a**–**c**) derivatives are spread over the whole molecule indicating very small participation for the substituents at position 3 of the thiophene ring fused with the cyclohexyl ring, while the LUMO display strong participation for these groups. The values of energy of both the HOMO and LUMO reflect this effect where the E_HOMO_ data indicate that all compounds have almost the same value, −7.22 eVm, while the E_LUMO_ values of the investigated compounds increase gradually and can be ordered as **6a** (−5.25) < **6b** (−5.32) > **6c** (−5.44).

The electronegative groups cause a decrease in the value of E_LUMO_ [52], and therefore, it could be concluded that the cyano group is the most electronegative, while the ester group is the least, in the (**6c**) and (**6a**) derivatives, respectively.

The ΔE_HOMO-LUMO_ data refer to the highest value of dye (**6c**), while the (**6b**) derivative has the lowest, 1.79 and 1.90 eV, respectively. This can be attributed to the increase in the mesomeric effect of substituents on the thiophene ring, which lead to a decrease in the E_g_. This enabled molecules to absorb more photons from sunlight beams [53]. Thus, dye (**6b**) with the lowest ΔE value is probably the most suitable for solar cell application.

#### 2.6.3. Mulliken Atomic Charges

The Mulliken atomic charges data of the investigated compounds show that the nitrogen atoms of the azo group have positive charges (Table S3, c.f. supplementary file S1). The charge value on the S atom of the pyridine ring fused with thiophene, S(18), confirms the high electron withdrawing character of the cyano group at 3-position compared to the amide and ester group, respectively. Moreover, the charge on the oxygen atom of carbonyl, O(26), for the amide group in (**6b**) and ester group in (**6a**) may be due to the higher electronegativity of the O than the N atoms, which are adjacent to the carbonyl group.

Fukui function analyses were used for evaluating the most active nucleophilic and electrophilic attack sites (Table S4, c.f. supplementary file S1). Compound (**6a**) shows that the most susceptible atoms for nucleophilic and electrophilic attacks are almost the same. Moreover, the data of dye (**6b**) exhibit that the susceptible sites for electrophilic attacks, i.e., electron donation, are S(18), O(26), N(13) and N(12) atoms. Furthermore, dye (**6c**) analysis shows the susceptible sites for electrophilic or nucleophilic attacks are almost the same but with a different order.

#### 2.6.4. Chemical Reactivity Descriptors

The chemical reactivity descriptors [50] such as electronegativity (*χ*), global hardness (η), softness (δ), and electrophilicity (*ω*) are discussed and calculated (c.f. supplementary file S1 and Table 4). As depicted in Table 4, dye (**6c**) has the higher Lewis acid character (*χ*), higher hardness (η) and higher electrophilicity index (*ω*) than the other two derivatives, which are almost equivalent.

## 3. Materials and Methods

### 3.1. General

All the chemicals and reagents were analytical grade or chemically pure and were supplied by Sigma Aldrich Co., Darmstadt, Germany. All of the micro, spectral analyses and electrochemical analyses were performed by the Micro Analytical Centers at Taif University, Taif, Saudi Arabia (IR spectra, UV-visible spectra (200–800 nm at RT), TEM and cyclic voltammetry) and at Mansoura University, Mansoura, Egypt (^1^H NMR, ^13^C NMR, MS, HRMS and computational studies). Methodology and instrumentations used were previously reported [54] and are explained in detail in the supplementary file S2.

### 3.2. Preparation and Characterization of Metal Nanoparticles (MNPs) and their Dye Conjugates

Metal nanoparticles were prepared in our laboratory in dimethyl formamide (DMF) according to the reported methods [36,55]. Characteristics of the synthesized metal nanoparticles (i.e., Au, Ag and Ru) were described in the literature [36,38] (c.f. supplementary file S2). For the preparation of the dyes/metal nanoparticle conjugates, the solid dyes were added to 5 mL of the freshly prepared hot MNPs colloidal solution in DMF. The cooled dye/MNP solutions were characterized using UV-Vis spectrophotometry.

### 3.3. Electrochemical Analysis

CV measurements were performed using a conventional three electrode cell configuration. Dyes (0.5 mM) were dissolved in DCM with TBAP (100 mM) as supporting electrolyte. CVs were recorded after background subtraction and IR compensation to minimize the double-layer charging current and solution resistance. CV data were measured at scan rate (0.02–5 *V/s*) in non-aqueous media at (25 ± 2) °C. All working solutions were thoroughly degassed and the experimental entries were maintained under nitrogen atmosphere. Methodology and instruments are explained in detail in the supplementary file S2.

### 3.4. Fabrication of Dye-Sensitized Solar Cell (DSSCs) and Photovoltaic Characterizations

Double-layer TiO_2_ photoelectrodes (10 + 5) μm were prepared and immersed into 0.5 mM of the synthesized compounds (**6a**–**c**) in ethanol, for 24 h at room temperature, and the Pt-counter electrodes were also prepared using reported techniques [11,56]. Both electrodes were sealed with 60 mm thick Surlyn (DuPont). Photoelectrochemical tests of the sealed cells were made by illuminating the dye-coated TiO_2_ film through the conducting glass support from the anode side with a solar simulator (WXS-155S-10) at AM 1.5 illuminations (light power of 100 mW cm^−2^, the equivalent of one sun at the surface of test cell). The detailed method is discussed in supplementary file S2.

### 3.5. Molecular Modeling

Geometrical optimization for the investigated compounds was performed using the Gaussian 09W program suite using the Becke3–Lee–Yang–Parr (B3LYP) exchange-correlation functional with standard 6-311++G (d,p) basis set [57,58,59,60]. Methodology and quantum chemical calculations are explained in detail in the supplementary file S1.

### 3.6. Synthesis and Characterization

#### 3.6.1. Synthesis of 2-amino-3-cyano-4-methylthiophene (**1**)

The compound was synthesized according to the reported method and found to be in agreement with literature [28]. Pink crystals; yield 95% (10.5 g); m.p.: 120–121 °C (Lit. 118–119 °C).

#### 3.6.2. Synthesis of 4-amino-6,7-dihydro-3-methyl-6-oxothieno[2,3-*b*]pyridine-5-carbonitrile (**3**)

A mixture of 2-amino-3-cyano-4-methylthiophene **1** (1.38 g, 10 mmol) and ethyl cyanoacetate (2.26 g, 20 mmol) was refluxed in sodium ethoxide (0.22 g Na/30 mL absolute ethanol, 10 mmol) for 2 h. The reaction mixture was left to cool and then poured onto ice-cold water containing HCl for neutralization. The solid product formed was collected using filtration, dried and recrystallized from ethanol to afford solid brown, yield 77.5% (1.59 g); m.p.: > 250 °C (Lit. > 360 °C [29]).

#### 3.6.3. General Procedure for the Synthesis of 4,5,6,7-tetrahydrobenzo[*b*]thiophenes (**4a**–**c**)

The cyclohexanone (0.98 g, 10 mmol) was mixed with active nitrile compounds (i.e., (**a**) ethyl cyanoacetate, 1.13 g; (**b**) cyanoacetamide, (**c**) 0.84 g; malononitrile, 0.66 g, 10 mmol), elemental sulfur (0.32 g, 10 mmol) and a catalytic amount of morpholine (2 mL) in ethanol (30 mL), according to the Gewald method [27]. The reaction mixture was then heated at 70 °C in a water bath (WB) for 2 h and left overnight to precipitate. The crystals of (**4a**–**c**) were collected with good yields and recrystallized from ethanol.

Ethyl 2-amino-4,5,6,7-tetrahydrobenzo[*b*]thiophene-3-carboxylate **4a**. Yellow needles; yield 67% (1.5 g); m.p.: 114–116 °C (Lit. 117–118 °C [61], 116–118 °C [31]).

2-Amino-4,5,6,7-tetrahydrobenzo[*b*]thiophene-3-carboxamide **4b**. Yellowish orange needles; yield 67% (1.5 g); m.p.: 180–181 °C (Lit. 180–181 °C [32], 185 °C [33]).

2-Amino-4,5,6,7-tetrahydrobenzo[*b*]thiophene-3-carbonitrile **4c**. Brown needles; yield 60% (1.1 g); m.p.: 140–142 °C (Lit. 147–148 °C [34]).

#### 3.6.4. General Procedure for Synthesis of thienopyridyl-azo-tetrahydrothiophene Derivatives (**6a**–**c**)

Molar quantities of thiophenes **4a**–**c** (2.25 g, 1.96 g, 1.78 g, respectively, 10 mmol) in (3 mL) conc. HCl and (5 mL) of an aqueous NaNO_2_ solution (10 mmol, 0.69 g) were mixed and well stirred at 0–5 °C for 30 min. The freshly prepared diazonium salts (**5a**–**c**) were added afterwards to (20 mL) of ethanolic solution for the coupling of component 2-aminothiazole **1** (2.05 g, 10 mmol) containing sodium acetate (3 g) with slow and continuous stirring at 0–5 °C for 2 h. The resulting products (**6a**–**c**) were collected, dried and recrystallized from MeOH/Dioxan.

Ethyl(*E*)-2-((4-amino-5-cyano-3-methyl-6-oxo-6,7-dihydrothieno[2,3-*b*]pyridin-2-yl)azo)-4,5,6,7-tetrahydrobenzo[*b*]thiophene-3-carboxylate **6a**. Brown crystals; yield 67.3% (2.96 g); m.p.: 109–110 °C. IR: ν_max_ = 3322–2936 (N–H_str._ and NH_2_), 2210 (CN), 1704 (CO_str._-), 1672 (CO_str._-), 1582 (N–H_bend._), 1450 (CH_2_) and 1396 (CH_3_) cm^−1^; ^1^H NMR: δ/ppm = 1.34 (t, *J* = 7.2 Hz, 3H, CH_3_), 1.83 (t, *J* = 4.4 Hz, 4H, 2CH_2_), 2.43 (s, 3H, CH_3_), 2.71 (t, *J* = 4.4 Hz, 2H, CH_2_), 2.83 (t, *J* = 4.4 Hz, 2H, CH_2_), 4.30 (q, *J* = 7.2 Hz, 2H, CH_2_), 6.41 (s, 2H, NH_2_) and 9.87 (s, 1H, NH); ^13^C NMR: δ/ppm = 8.2, 13.8, 22.8 (3C), 23.7, 60.4, 79.6, 110.1, 112.6, 115.1, 125.9, 127.3, 129.5, 133.2, 137.4, 159.6, 165.7, 168.1, 173.8; MS (*m*/*z*, %): 441 (M^+^, 63.4); HRMS calc. for C_20_H_19_N_5_O_3_S_2_: 441.0929, found: 441.0911.

(*E*)-2-((4-Amino-5-cyano-3-methyl-6-oxo-6,7-dihydrothieno[2,3-*b*]pyridin-2-yl)azo)-4,5,6,7-tetrahydrobenzo[*b*]thiophene-3-carboxamide **6b**. Reddish brown crystals; yield 96% (3.9 g); m.p.: 172–174 °C. IR: ν_max_ = 3324–2935 (N–H_str._ and NH_2_), 2209 (CN), 1664 (CO_str._-), 1637 (CO_str_.- amidic),1582 (N–H_bend._), 1449 (CH_2_) and 1394 (CH_3_) cm^−1^; ^1^H NMR: δ/ppm = 1.83 (t, *J* = 4.4 Hz, 4H, 2CH_2_), 2.59 (s, 3H, CH_3_), 2.66 (t, *J* = 4.4 Hz, 2H, CH_2_), 2.83 (t, *J* = 4.4 Hz, 2H, CH_2_), 6.35 (s, 2H, NH_2_) 7.81 (s, 2H, NH_2_) and 10.96 (s, 1H, NH); ^13^C NMR: δ/ppm = 7.9, 22.9 (2C), 23.1, 24.2, 80.1, 109.8, 113.3, 115.2, 116.4, 124.9, 133.2, 135.4, 138.5, 165.8, 167.5, 168.1, 176.0; MS (*m*/*z*, %): 412 (M^+^, 25.1); HRMS calc. for C_18_H_16_N_6_O_2_S_2_: 412.0776, found: 412.0761.

(*E*)-4-Amino-2-((3-cyano-4,5,6,7-tetrahydrobenzo[*b*]thiophen-2-yl)azo)-3-methyl-6-oxo-6,7-dihydrothieno[2,3-*b*]pyridine-5-carbonitrile **6c**. Dark Brown crystals; yield 74.6% (2.9 g); m.p.:148–149 °C. IR: ν_max_ = 3327–2935 (N–H_str._ and NH_2_), 2209 (CN), 1664 (CO_str._-), 1582 (N–H_bend._), 1448 (CH_2_) and 1396 (CH_3_) cm^−1^; ^1^H NMR: δ/ppm = 1.73 (t, *J* = 4.4 Hz, 4H, 2CH_2_), 2.51 (s, 3H, CH_3_), 2.72 (t, *J* = 4.4 Hz, 2H, CH_2_), 2.89 (t, *J* = 4.4 Hz, 2H, CH_2_), 6.56 (s, 2H, NH_2_) and 9.11 (s, 1H, NH); ^13^C NMR: δ/ppm = 8.1, 22.7 (2C), 23.4, 23.9, 81.0, 102.3, 110.1, 113.5, 114.9, 115.2, 133.5, 134.1, 135.1, 140.3, 166.0, 167.8, 176.0; MS (*m*/*z*, %): 394 (M^+^, 13.1); HRMS calc. for C_18_H_14_N_6_OS_2_: 394.0671, found: 394.0660.

## 4. Conclusions

Our work demonstrates that the introduction of a substituted thiophene segment is of great benefit as a D-π-A organic sensitizer for solar cell applications. Thus, three novel diazenyl pyridothiophene derivatives (**6a**–**c**) were synthesized as the result of the diazo coupling reaction of 2-amino-4,5,6,7-tetrahydrobenzo[*b*]thiophenes (**4a**–**c**) with 4-amino-6,7-dihydro-3-methyl-6-oxothieno[2,3-*b*]pyridine-5-carbonitrile (**3**). Their chemical structures are established on the basis of their spectral data. The substituent groups (i.e., COOEt, CONH_2_ and CN) are conjugated with the whole molecule, leading to light-harvesting capability of the dye and, more importantly, quite a smooth electron injection process. The effect of the anchoring groups is studied based on the band gap energy (E_g_) of their FMOs. Spectroscopy and electrochemical analysis confirmed that the keto acceptor of dye (**6b**) is a relatively stronger acceptor and improved the photovoltaic performance compared with other substituents. The modeling study is in good agreement with the practical findings. Plasmon resonance employing noble metals (i.e., gold, silver and ruthenium) has been recently introduced to the DSSCs using electrostatic interaction with the synthesized diazenyl thieno-pyridyl dyes, where the localized surface plasmon resonance phenomena of metal nanoparticles enhance the light harvesting efficiency. The conjugation with silver nanoparticles (**6b**/AgNPs) exhibits the best results over the studied alternatives. This finding is supported by the data measured and calculated of the HOMO-LUMO band gap energy. The introduction of stronger accepting and donating groups and increasing the planarity of the system using a triple bond bridge will provide a powerful strategy for the development of thiophene based highly efficient D-π-A organic sensitizer in the future.

## Figures and Tables

**Table 1:** Electrochemical analysis data of the synthesized dyes (**6a**–**c**).

Dye	E _HOMO_ (eV)	E _LUMO_ (eV)	Eg (eV)
**6a**	−3.30	−3.55	0.25
**6b**	−3.40	−3.20	0.20
**6c**	−3.13	−3.73	0.60

**Table 2:** Electrochemical analysis data of the synthesized dye (**6b**) conjugated with Au, Ag and Ru NPs.

Dye/MNPs	E _HOMO_ (eV)	E _LUMO_ (eV)	Eg (eV)
**6b**/AuNPs	−3.10	−3.43	0.33
**6b**/AgNPs	−3.31	−3.40	0.09
**6b**/RuNPs	−2.91	−3.43	0.52

**Table 3:** *J-V* characteristics of solar devices based on the synthesized dyes (**6a**‒**c**) and (**6b**/NPs) conjugates.

Dye	*J_SC_* (mA.cm^−2^)	*V_OC_* (V)	*FF*	η *(%)*
**6a**	4.309 × 10^−2^	0.054	0.27	6.17 × 10^−4^
**6b**	4.313 × 10^−2^	0.141	0.53	3.24 × 10^−3^
**6c**	3.243 × 10^−2^	0.102	0.38	1.27 × 10^−3^
**6b**/Au-NPs	4.065 × 10^−2^	0.182	0.38	2.79 × 10^−3^
**6b**/Ru-NPs	5.561 × 10^−2^	0.197	0.39	4.16 × 10^−3^
**6b**/Ag-NPs	1.136 × 10^−1^	0.436	0.57	2.82 × 10^−2^

*J_SC_*, short-circuit photocurrent density; *V_OC_*, open-circuit photovoltage; *FF*, fill factor; η, total power conversion efficiency.

**Table 4:** Chemical reactivity descriptors (eV) and dipole moment (Debye) of investigated dyes (**6a**–**c**)**.**

	6a	6b	6c
E_HOMO_	−7.23	−7.22	−7.23
E_LUMO_	−5.29	−5.32	−5.44
ΔE	1.94	1.90	1.79
χ	6.26	6.27	6.34
η	0.97	0.95	0.89
δ	1.03	1.05	1.12
ω	20.17	20.71	22.49
I	7.23	7.22	7.23
A	5.29	5.32	5.44
μ_total_	10.09	8.32	11.54
μ_x_	9.69	−7.82	10.43
μ_y_	−1.80	−2.53	−4.93
μ_z_	2.16	−1.28	−0.26

## Supplementary Materials

The following are available online, Modeling S1: Modeling study, Experimental S2: Experimental.
