# Supplementary material for: Design, Molecular Modeling and Synthesis of Metal-Free Sensitizers of Thieno Pyridine Dyes as Light-Harvesting Materials with Efficiency Improvement Using Plasmonic Nanoparticles
Source: Molecules. 2020 Apr 15;25(8):1813. doi: 10.3390/molecules25081813 (PMC7221727; doi:10.3390/molecules25081813)
Supplement: Supplementary file 1 [file molecules-25-01813-s001.pdf]

Design, molecular modeling and synthesis of metal-free sensitizers of thieno pyridine dyes as light-harvesting materials with efficiency improvement by plasmonic nanoparticles

Supplementary file S1

Computational study

1. Experimental

1.1. Methods and Instrumentation

Quantum chemical calculations are used mainly for geometrical optimization, predicting the Mulliken atomic charges, calculating the frontier molecular orbitals and determining the shape and energy for the compounds under investigation with the aid of Gaussian 03W suite program at Semi-empirical PM6 level [1-3] using the Becke3–Lee–Yang–Parr (B3LYP) exchange-correlation functional [4-6] with standard 6-311++G (d,p) basis set. The geometrical structure is among the important factors governing the chemical and biological activities [7]. The Mulliken atomic charge is also an important factor that affecting the reactivity towards electrophilic or nucleophilic reaction [8]. The frontier molecular orbitals (FMO's) are simply the highest occupied and lowest unoccupied molecular orbital (HOMO and LUMO). They are used to find frontier electron density, therfore predicting the most reactive positions in the molecule [9]. Due to exhibit excellent understanding of electronic, spectral and structural properties in addition to coincide with the experimental data of organic and inorganic compounds, the density functional theory (DFT) method have gained a great attention in last two decades [10]. Fukui indices have been calculated using DMol3 module of Materials Studio package (from Accelrys Inc.) [11]. The gradient-corrected functional method (GGA) with a double numeric plus polarization (DNP) basis set (version 3.5) and a Becke One Parameter (BOP) functional [12]have been used.

2. Results and Discussion

2.1. Molecular Geometry

The dihedral angles data of DFT optimized structures, shown in Table S1

Table S1. Selected DFT calculated dihedral angles of investigated compounds.

Dihedral angle	6a (°)	6b (°)	6c (°)

C_{thio}-C_{thio}-C_{thio}-S (Py)	2.17	-5.75	-4.80
C_{thio}-C_{thio}-C_{thio}-S (Py)	0.66	-3.13	4.42
C_{thio}-C_{thio}-C_{thio}-S (Cyclo)	-2.00	5.79	-0.91
C_{thio}-C_{thio}-C_{thio}-S (Cyclo)	0.81	-0.42	-3.21
C_{thio}-C_{thio}-C_{thio}-C_{thio} (Py)	-1.82	5.67	-7.44
C_{thio}-C_{thio}-C_{thio}-C_{thio} (Cyclo)	0.71	-3.29	2.62
C_{thio}-N_{azo}-N_{azo}-C_{thio}	174.81	-171.95	-179.11
N_{azo}-N_{azo}-C_{thio}-S (Py)	-169.46	-149.89	-138.98
N_{azo}-N_{azo}-C_{thio}-S (Cyclo)	-155.68	106.04	150.87
S-C_{thio}-C_{cyc}-C_{cyc}	164.96	165.15	164.64
S-C_{thio}-C_{thio}-C_{cyc}	176.92	179.02	-179.71
C_{thio}-C_{cyc}-C_{cyc}-C_{cyc}	42.92	45.27	45.02
C_{thio}-C_{thio}-C_{cyc}-C_{cyc}	-12.97	-14.49	-16.04
C_{thio}-C_{thio}-CO-O	-132.24	-140.92	110.73
C_{thio}-C_{thio}-CO-O_{Et}	46.59	39.62	
S-C_{thio}-C_{thio}-CO	-171.39	-178.87	-179.60
S-C_{thio}-C_{thio}-C_{NH2py}	175.80	173.01	-169.30
CO_{py}-C_{CNpy}-C_{NH2py}-NH_2	121.05	142.38	-179.54
S-C_{thio}-NH_{py}-H_{NH}	-0.79	2.02	-4.46
S-C_{thio}-NH_{py}-CO_{py}	-179.70	-177.70	168.90
C_{thio}-NH_{py}-CO_{py}-O	178.92	179.54	178.86
C_{thio}-C_{thio}-C_{NH2py}-C_{CNpy}	10.03	3.10	-4.90
C_{thio}-NH_{py}-CO_{py}-C_{CNpy}	-1.71	5.69	3.58
NH_{py}-CO_{py}-C_{CNpy}-CN	-119.95	124.55	175.86

The DFT bond length data for selected bonds, in comparison with x-ray single crystal for analogue compounds listed in Table S2.

Table S2. Selected DFT bond length (Å) for compounds **(6a-c)** in comparison with x-ray data [13-17]

**Bond**	**6a**	**6b**	**6c**	**x-ray**
C-$S_{(cyc)}$	1.64	1.67	1.63	1.62-1.76
C-$S_{(cyc)}$	1.73	1.72	1.74	1.62-1.76
C-$S_{(py)}$	1.78	1.81	1.66	1.62-1.76
C-$S_{(py)}$	1.73	1.70	1.81	1.62-1.76
N-$N_{(azo)}$	1.24	1.24	1.25	1.18-1.31
C_{thio}-N_{azo}	1.46	1.41	1.44	1.36-1.50
C_{thio}-N_{azo}	1.46	1.45	1.47	1.36-1.50
N-$H_{(py)}$	1.01	1.01	1.02	0.99-1.03
H_2N-$H_{(py)}$	1.05	1.05	1.00	1.01-1.06
H_2N-$H_{(py)}$	1.05	1.05	1.01	1.01-1.06
C_{py}-NH_2	1.47	1.44	1.35	1.41-1.47
C-$N_{(py)}$	1.16	1.16	1.16	1.13-1.18
C=$O_{(py)}$	1.22	1.22	1.22	1.19-1.25
C=$O_{(ester)}$	1.35			1.33-1.36
C_{thio}-CO_{ester}	1.53			

CO-O_{Et}	1.21			
C=$O_{(amide)}$		1.22		1.19-1.25
C_{thio}-$CO_{(amide)}$		1.57		
CO-$NH_{2(amide)}$		1.36		
H_2N-$H_{(amide)}$		1.01		1.01-1.06
H_2N-$H_{(amide)}$		1.02		1.01-1.06
C-$N_{(thio)}$			1.14	1.13-1.18

Finally, dipole moment (μ_{total}) reflects the molecular charge distribution and is given as a vector in three dimensions, μ_x, μ_y, and μ_z. Therefore, it can be used as descriptor to depict the charge movement across the molecule. Direction of the dipole moment vector in a molecule depends on the centers of positive and negative charges. If geometries are symmetric, the dipole moments components are zero [18]. The data of the investigated compounds was shown in Table S3.

Table S3. Chemical reactivity descriptors (eV) and dipole moment (Debye) of investigated compounds

	**6a**	**6b**	**6c**
E_{HOMO}	-7.23	-7.22	-7.23
E_{LUMO}	-5.29	-5.32	-5.44
**ΔE**	1.94	1.90	1.79
χ	6.26	6.27	6.34
η	0.97	0.95	0.89
δ	1.03	1.05	1.12
ω	20.17	20.71	22.49
**I**	7.23	7.22	7.23
**A**	5.29	5.32	5.44
μ_{total}	10.09	8.32	11.54
μ_x	9.69	-7.82	10.43
μ_y	-1.80	-2.53	-4.93
μ_z	2.16	-1.28	-0.26

2.2. *Frontier molecular orbitals*

The highest occupied and lowest unoccupied molecular orbitals, HOMO and LUMO, respectively, are called the frontier molecular orbitals and they can determine the way in which the molecule interacts with other species. The HOMO and LUMO energies indicate the molecules ability to donate and accept an electron, respectively. The energies of these two molecular orbitals were calculated and presented in Table S3. The HOMO-LUMO gap, ΔE = E_{HOMO}–E_{LUMO}, used for predicting the

relationship of the chemical structure with electronic properties such as frontier electron densityand chemical stability; and explains the ultimate charge transfer in the molecule [19, 20].

The graphical representation of molecular orbitals can provide insight into the aromaticity and lone pair. The positive and negative phase of wave-functions are represented in red and green color, respectively [21].

The electronegative groups cause a decrease in the value of E_{LUMO} [13]. For solar cell applications, molecules have small $\Delta E_{HOMO-LUMO}$ can absorb more photons from sun light beam [22].

2.3. *Mulliken atomic charges*

The Mulliken atomic charges data of investigated compounds was shown in Table 4.

Table 4. Selected Mulliken atomic charges for investigated compounds.

6a		6b		6c	
**Atoms**	**Charge**	**Atoms**	**Charge**	**Atoms**	**Charge**
N(12)	0.241	N(11)	-0.147	N(11)	-0.215
N(13)	0.261	N(12)	0.155	N(12)	0.161
O(25)	0.148	N(13)	0.145	N(13)	0.194
N(11)	-0.231	S(18)	-0.289	S(18)	-0.237
S(18)	-0.371	O(24)	-0.306	O(23)	-0.304
O(24)	-0.309	N(25)	-0.358	N(25)	-0.218
O(26)	-0.134	O(26)	-0.298	N(27)	-0.168
N(30)	-0.218	N(28)	-0.227	S(4)	0.055
S(4)	-0.138	S(4)	-0.145	N(7)	-0.181
N(7)	-0.154	N(7)	-0.181		

2.3.1. *Chemical reactivity descriptors*

The E_{HOMO} and E_{LUMO} are related to the ionization potential (I) and the electron affinity (A) of molecules by the following relations $I = -E_{HOMO}$ and $A = -E_{LUMO}$. Besides the traditional reactivity descriptors, such as frontier molecular orbitals, there are certain other chemical reactivity descriptors such as electronegativity (χ), global hardness (η), softness (δ), and electrophilicity (ω) which can be calculated from HOMO–LUMO. The electronegativity (χ) is a measure to the molecule power to attract electrons and calculated as follows [19].

$$\chi = -\frac{1}{2}(E_{HOMO} + E_{LUMO}) \qquad (1)$$

$$\eta = -\frac{1}{2}(E_{HOMO} - E_{LUMO}) \qquad (2)$$

$$\delta = \frac{1}{\eta} \qquad (3)$$

$$\omega = \frac{\chi^2}{2\eta} \qquad (4)$$

The large χ values characterize Lewis acid while small (χ) values are for bases. Global hardness (η) is a measure of the charge transfer resistance while the global softness (δ) describes the capacity of a molecule to receive electrons and is equal to reciprocal of global hardness [19]. Soft molecules, small energy gap, are more reactive than hard ones because they could easily offer electrons to an acceptor.

The electrophilicity (ω) is a measure of energy lowering due to the maximal electron flow between the donor, HOMO, and the acceptor, LUMO, and can be calculated from the electronegativity and chemical hardness.

Finally, Fukui function analyses are very useful to evaluate the most active sites for nucleophilic and electrophilic attacks. The Fukui indices for investigated compounds are calculated using the following equations, to explore the reactivity of each atom in these molecules [23-25] (Table 4), where $q_k(N)$, $q_k(N+1)$and $q_k(N-1)$ are the atomic charges of the systems with N, N+1 and N-1 electrons, respectively [26].

$$f_k^+ = q_k(N+1) - q_k(N) \qquad (5)$$

$$f_k^- = q_k(N) - q_k(N-1) \qquad (6)$$

References

[1] J.J.P. Stewart, Optimization of parameters for semiempirical methods V: Modification of NDDO approximations and application to 70 elements, J. Mol. Model. 13(12) (2007) 1173-1213.

[2] J. Pople, M. Frisch, G. Trucks, H. Schlegel, G. Scuseria, Gaussian, Inc., Wallingford CT, 2004 Gaussian 03W (Revision C. 01), Gaussian, Inc., Wallingford CT (2003).

[3] R. Dennington, T. Keith, J. Millam, GaussView, Semichem Inc., Shawnee Mission, KS, Semichem Inc., USA, 2009.

[4] A.D. Becke, Density-functional thermochemistry. III. The role of exact exchange, J. Chem. Phys. 98(7) (1993) 5648-5652.

[5] C. Lee, W. Yang, R.G. Parr, Development of the Colle-Salvetti correlation-energy formula into a functional of the electron density, Phys. Rev. B 37(2) (1988) 785.

[6] J.P. Perdew, Y. Wang, Pair-distribution function and its coupling-constant average for the spin-polarized electron gas, Phys. Rev. B 46(20) (1992) 12947.

[7] M. Karelson, V.S. Lobanov, A.R. Katritzky, Quantum-chemical descriptors in QSAR/QSPR studies, Chem. Rev. 96(3) (1996) 1027-1044.

[8] L.R. Domingo, P. Pérez, J.A. Sáez, Understanding the local reactivity in polar organic reactions through electrophilic and nucleophilic Parr functions, RSC Advances 3(5) (2013) 1486-1494.

[9] K. Fukui, T. Yonezawa, H. Shingu, A molecular orbital theory of reactivity in aromatic hydrocarbons, J. Chem. Phys. 20(4) (1952) 722-725.

[10] F. Neese, Prediction of molecular properties and molecular spectroscopy with density functional theory: From fundamental theory to exchange-coupling, Coord. Chem. Rev. 253(5-6) (2009) 526-563.

[11] Materials Studio package, BIOVIA, Dassault Systèmes, San Diego, USA, 2017.

[12] B. Delley, Ground-state enthalpies: evaluation of electronic structure approaches with emphasis on the density functional method, J. Phys. Chem. A 110(50) (2006) 13632-13639.

[13] R. Amorim, M.D.F. de Meneses, J.C. Borges, L.C. da Silva Pinheiro, L.A. Caldas, C.C. Cirne-Santos, M.V.P. de Mello, A.M.T. de Souza, H.C. Castro, I.C.N. de Palmer Paixao, Thieno [2, 3-b] pyridine derivatives: a new class of antiviral drugs against Mayaro virus, Arch. Virol. 162(6) (2017) 1577-1587.

[14] Y.N. Mabkhot, A. Barakat, S.M. Soliman, T.T. El-Idreesy, H.A. Ghabbour, S.S. Al-Showiman, Synthesis, characterization and computational studies of a novel thieno [2, 3-b] thiophene derivative, J. Mol. Struct. 1130 (2017) 62-70.

[15] H. Chen, M.-G. Liu, Synthesis, characterization and crystal structure of heterocyclic tetrahydropyrido [4′, 3′: 4, 5] thieno [2, 3-d] pyrimidinone derivatives via sequential aza-Wittig/base catalyzed cyclization, J. Mol. Struct. 1180 (2019) 31-40.

[16] V. Khojasteh, A. Kakanejadifard, A. Zabardasti, F. Azarbani, Spectral, structural, solvatochromism, biological and computational investigation of some new azo–azomethines containing N-alkylpyridinium salts, J. Mol. Struct. 1175 (2019) 261-268.

[17] P. Goszczycki, K. Stadnicka, M.Z. Brela, J. Grolik, K. Ostrowska, Synthesis, crystal structures, and optical properties of the π-π interacting pyrrolo [2, 3-b] quinoxaline derivatives containing 2-thienyl substituent, J. Mol. Struct. 1146 (2017) 337-346.

[18] A. Datta, Role of metal ions (M= Li+, Na+, and K+) and pore sizes (Crown-4, Crown-5, and Crown-6) on linear and nonlinear optical properties: New materials for optical birefringence, The Journal of Physical Chemistry C 113(8) (2009) 3339-3344.

[19] S. Xavier, S. Periandy, S. Ramalingam, NBO, conformational, NLO, HOMO–LUMO, NMR and electronic spectral study on 1-phenyl-1-propanol by quantum computational methods, Spectrochim. Acta A 137 (2015) 306-320.

[20] M.M. Makhlouf, A.S. Radwan, B. Ghazal, Experimental and DFT insights into molecular structure and optical properties of new chalcones as promising photosensitizers towards solar cell applications, Appl. Surf. Sci. 452 (2018) 337-351.

[21] V. Arjunan, P. Balamourougane, M. Kalaivani, A. Raj, S. Mohan, Experimental and theoretical quantum chemical investigations of 8-hydroxy-5-nitroquinoline, Spectrochim. Acta A 96 (2012) 506-516.

[22] Ö. Tayfuroğlu, F.A. Kılıçarslan, G.Y. Atmaca, A. Erdoğmuş, Synthesis, characterization of new phthalocyanines and investigation of photophysical, photochemical properties and theoretical studies, Journal of Porphyrins and Phthalocyanines 22(01n03) (2018) 250-265.

[23] L.O. Olasunkanmi, I.B. Obot, E.E. Ebenso, Adsorption and corrosion inhibition properties of N-{n-[1-R-5-(quinoxalin-6-yl)-4, 5-dihydropyrazol-3-yl] phenyl} methanesulfonamides on mild steel in 1 M HCl: experimental and theoretical studies, RSC Advances 6(90) (2016) 86782-86797.

[24] Z. El Adnani, M. Mcharfi, M. Sfaira, M. Benzakour, A. Benjelloun, M.E. Touhami, DFT theoretical study of 7-R-3methylquinoxalin-2 (1H)-thiones (RH; CH3; Cl) as corrosion inhibitors in hydrochloric acid, Corrosion Science 68 (2013) 223-230.

[25] H. Mi, G. Xiao, X. Chen, Theoretical evaluation of corrosion inhibition performance of three antipyrine compounds, Computational and Theoretical Chemistry 1072 (2015) 7-14.

[26] M. Messali, M. Larouj, H. Lgaz, N. Rezki, F. Al-Blewi, M. Aouad, A. Chaouiki, R. Salghi, I.-M. Chung, A new schiff base derivative as an effective corrosion inhibitor for mild steel in acidic media: Experimental and computer simulations studies, J. Mol. Struct. 1168 (2018) 39-48.

Design, molecular modeling and synthesis of metal-free sensitizers of thieno pyridine dyes as light-harvesting materials with efficiency improvement by plasmonic nanoparticles

Supplementary file S2

Experimental

Micro and spectroscopic analysis

All of the corrected melting points in degree centigrade are measured using a Stuart SMP 20 melting point apparatus (Bibby Scientific Ltd., Staffordshire, UK). The infrared spectra were recorded on a PerkinElmer Alpha platinum-ATR spectrometer, the ^{1}H NMR (300 MHz) and ^{13}C NMR (75 MHz) spectra are recorded on a Varian Mercury VXR-300 spectrometer (Varian Inc., Palo Alto, CA, USA) and the chemical shifts were related to that of the solvent DMSO-d_6 using TMS as an internal standard. The EI-Ms analysis and the high resolution MS spectra (HRMS) are acquired on TRACE GC Ultra Gas Chromatographs–mass spectrometry, coupled with Thermo mass spectrometer detector-ISQ Single Quadrupole Mass Spectrometer (Thermo Scientific Corp., Waltham, MA, USA) and are obtained by electron ionization (EI) at 70 eV, using a spectral range of *m*/*z* 50-1000. UV-visible spectra are measured using Perkin Elmer spectrometer (Perkin Elmer Co., Shelton, UK).

Synthesis and characterization of Metals nanoparticles (MNPs)

Metal nanoparticles are prepared in our laboratory in dimethyl formamide (DMF) according to the reported methods [1, 2]. The color changed from yellow to colorless then to wine red to afford gold nanoparticles' solution (AuNPs), while from colorless to yellow to afford silver nanoparticles' solution (AgNPs) and from dark red to light yellow then turned to dark brown to afford ruthenium nanoparticles' solution (RuNPs). The surface properties, morphological features and size of MNPs are analyzed using UV–Vis spectrophotometry, TEM and nanophox particle analyses. Characteristics of the synthesized metal nanoparticles (i.e. Au, Ag and Ru) are described by the literature [1, 3]. The surface properties of metals nanoparticles (MNPs) are analyzed using a transmission electron microscope (1200 EX, JOEL Inc, Peabody, MA, USA).

Electrochemical analysis

Cyclic voltammetry measurements were performed using a conventional three electrode cell configuration; a glassy carbon working electrode, a platinum wire as a counter electrode and an Ag/AgCl as reference electrode (calibrated against ferrocene/ferrocenium couple, linked to an EG and G model 283 Potentiostat (Princeton Applied Research, Oak Ridge, Tennessee, USA). Dyes (0.5 mM) are dissolved in DCM with TBAP (0.1 M) as supporting electrolyte. Cyclic voltammograms are recorded after background subtraction and IR compensation to minimize double-layer charging current and solution resistance. Cyclic voltammetric data are obtained at scan rate ranging from 0.02 to 5 V/s in non- aqueous media at (25 ± 2) °C. All working solutions are thoroughly degassed with oxygen free nitrogen, and a nitrogen atmosphere is maintained above the solution throughout experimental studies.

Molecular modeling

Quantum chemical calculations for investigated compounds are used to optimize the geometry by Gaussian 09W suite program using the Becke3–Lee–Yang–Parr (B3LYP) exchange-correlation functional with standard 6-311++G (d,p) basis set [4-7]. The obtained geometrical optimized structures and frontier molecular orbitals (FMO's) are visually inspected using the GaussView program [8]. Fukui indices have been calculated using DMol3 module of Materials Studio package (Accelrys Inc., San Diego, CA, USA) [9]. The gradient-corrected functional method (GGA) with a double numeric plus polarization (DNP) basis set (version 3.5) and a Becke One Parameter (BOP) functional have been used [10].

Fabrication of the dye-sensitized solar cells (DSSCs)

A double-layer TiO_2 photoelectrodes (10+ 5) μm in thickness with 10 μm thick nanoporous layer and 5 μm thick scattering layer (area: 0.25 cm^2) are prepared, rinsed with water and ethanol, sintered at 500 °C for 30 min and exposed to an O_2 plasma with the guide of the reported techniques [11, 12]. Then they are immersed into a 0.5 mM photosensitizer solution (ethanol) containing synthesized compounds (**6a-c)**, for 24 h at room temperature. Pt-counter electrodes are prepared by placing a drop of an H_2PtCl_6 solution (0.02M in ethanol) on the fluorine doped tin oxide (FTO) glass with heating treatment at 400 °C for 15 min. In this work, a mixture of 0.5M 1-butyl-3-methylimidiazolium iodide, 0.10M LiI, 0.05M I_2, and 0.5M tert-butylpyridine in acetonitrile is used as the redox electrolyte [13]. The dye-adsorbed TiO_2 electrodes and the Pt counter electrodes are assembled into a sealed sandwich-type cell by heating at 80 °C, using a hot-melt ionomer film Surlyn (DuPont) as a sealant and spacer between the electrodes [13, 14]. A drop of the electrolyte solution is placed in the drilled hole of the counter electrode and was driven into the cell via vacuum backfilling. Finally, the hole is sealed using additional Surlyn and a cover glass (0.1 mm thickness). Both the photosensitizer-adsorbed TiO_2 electrode and the Pt-counter electrode are sealed with 60 mm thick Surlyn. Photoelectrochemical tests of sealed cells are made by illuminating the dye-coated TiO_2 film through the conducting glass support from the anode side with a solar simulator (WXS-155S-10) at AM 1.5 illuminations (light power of 100 mW cm^{-2}, the equivalent of one Sun at the surface of test cell.).

Experimental figures

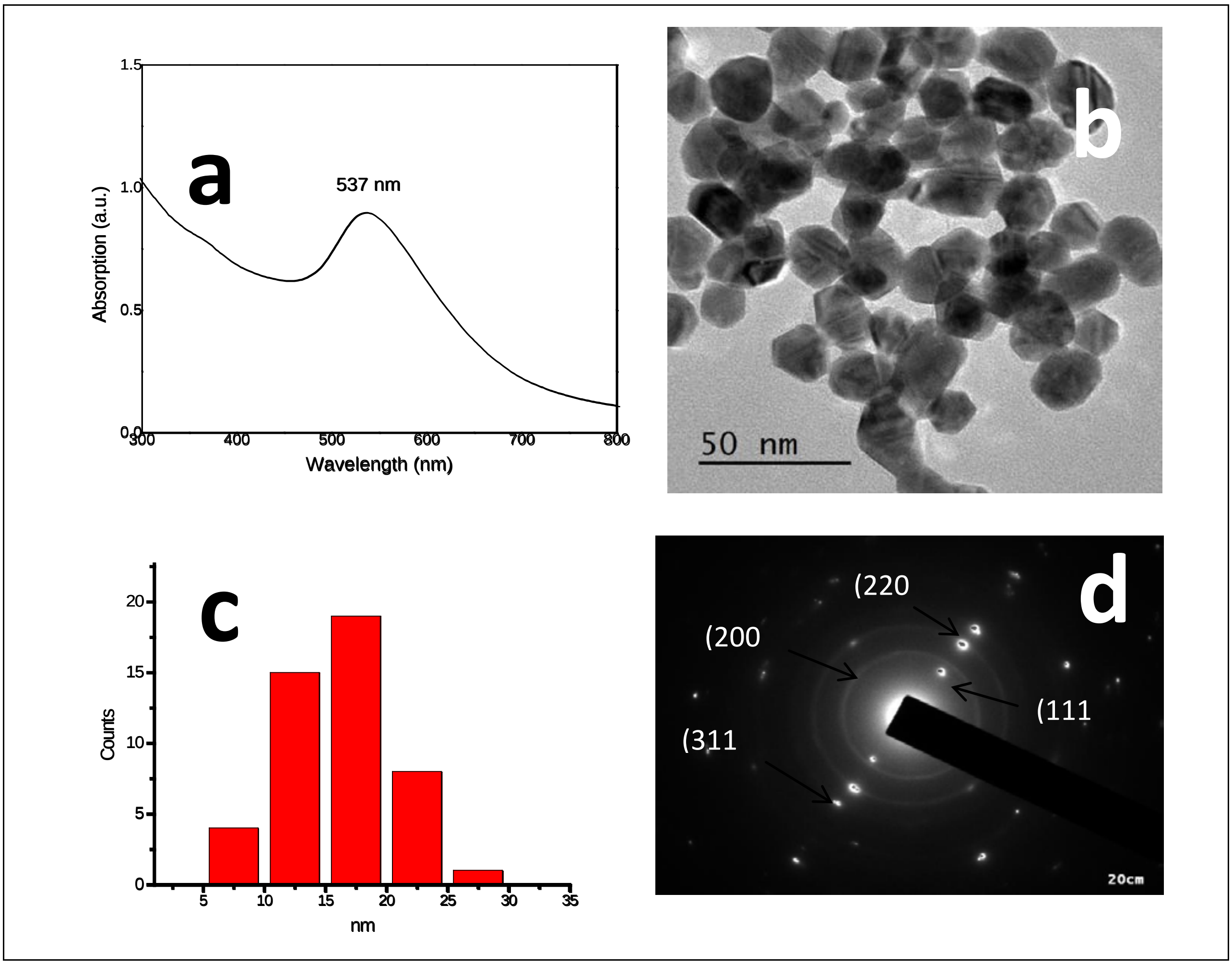

Fig. S1 (**a**) UV-Vis absorbance of AuNPs prepared in DMSO; (b) HRTEM images of AuNPs (**c**) particle size distribution of AuNPs; (d) selected area electron diffraction (SAED).

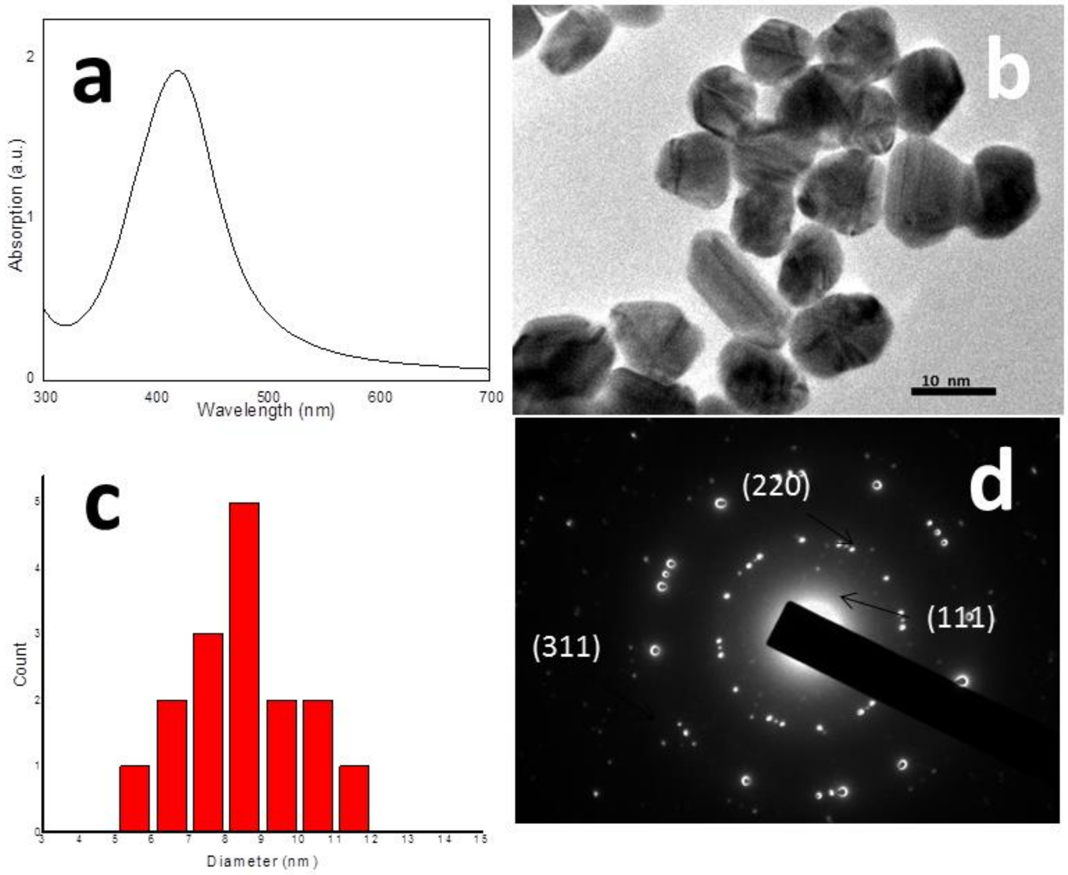

Fig. S2 (**a**) UV-Vis absorbance of AgNPs prepared in DMSO; (b) HRTEM images of AgNPs (**c**) particle size distribution of AgNPs; (d) selected area electron diffraction (SAED).

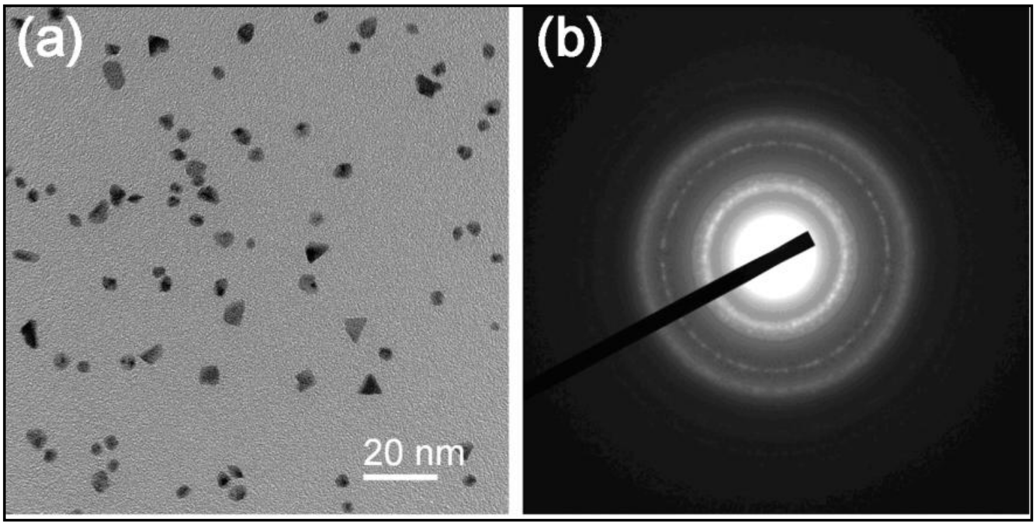

Fig. S3 (a) HRTEM images of RuNPs; (**b**) selected area electron diffraction (SAED).

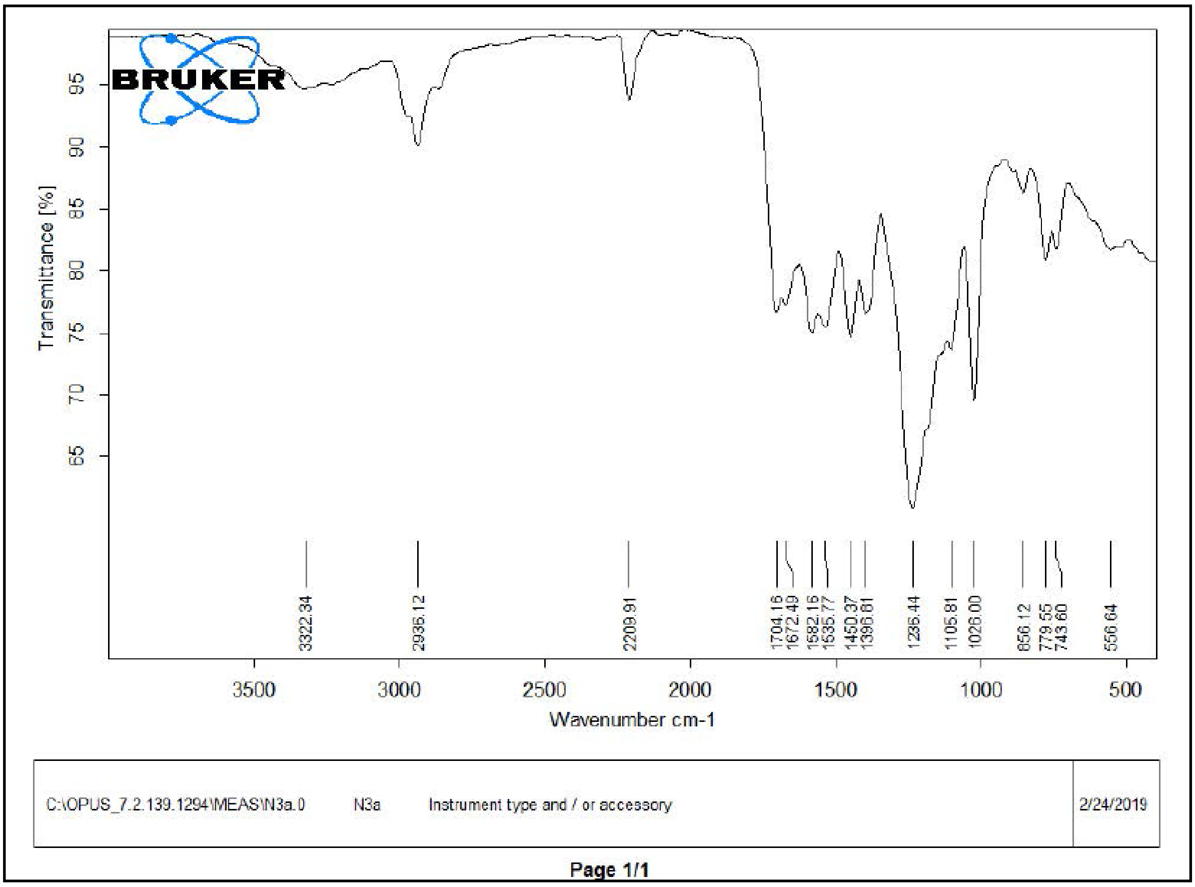

Fig.S4. IR spectra of compound **(6a)**.

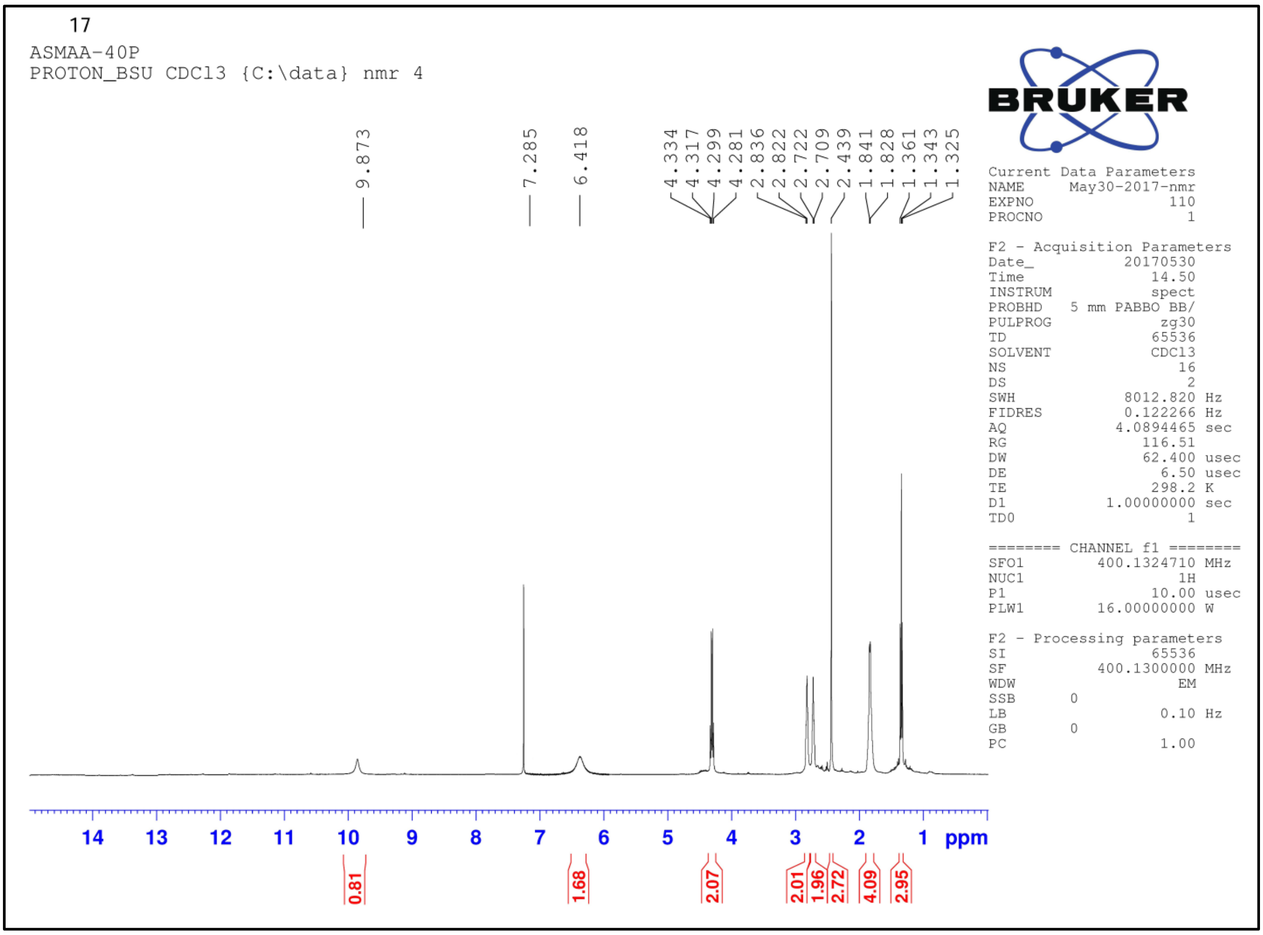

Fig.S5. ^{1}H NMR spectrum of compound (**6a**)

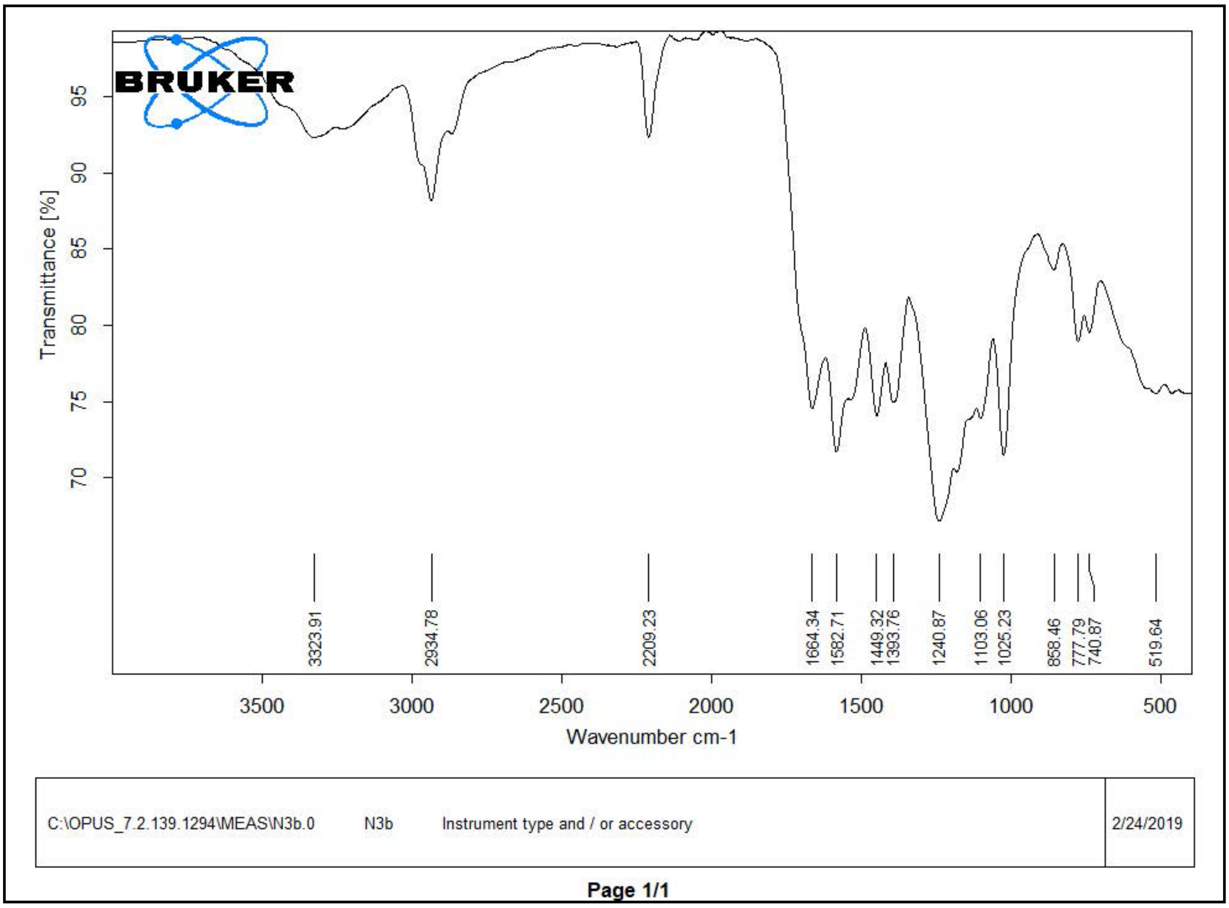

Fig. S6. IR spectra of compound **(6b)**.

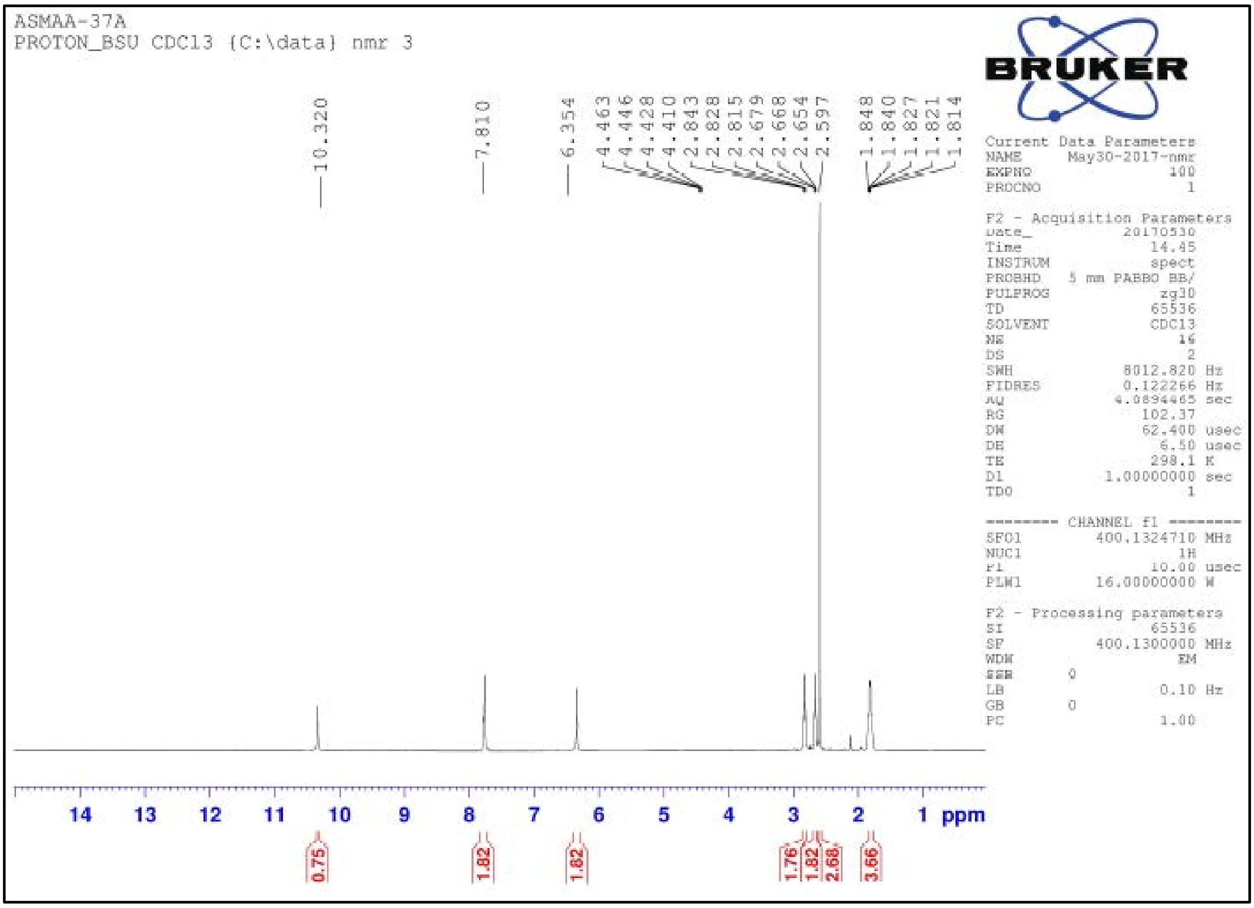

Fig. S7.^{1}H NMR spectrum of compound **(6b)**.

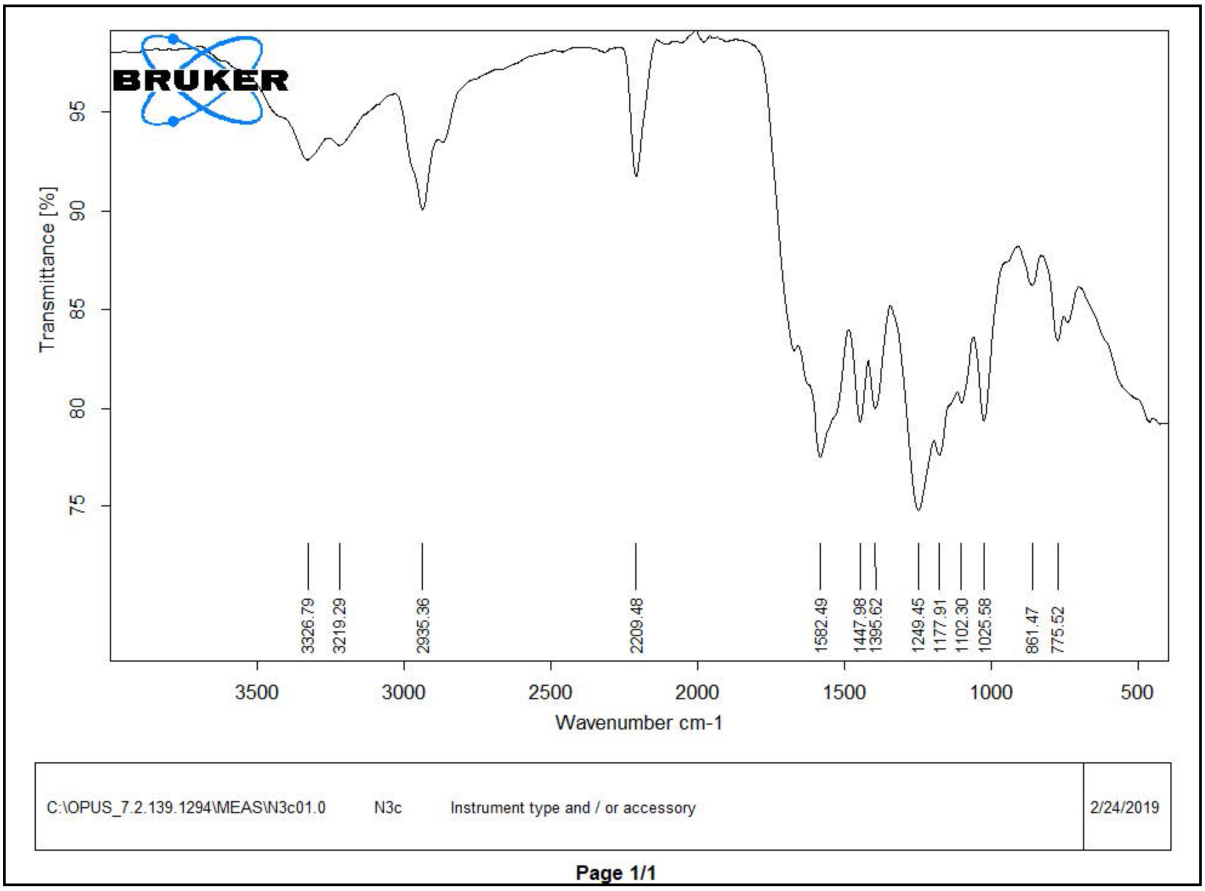

Fig. S8. IR spectra of compound **(6c)**.

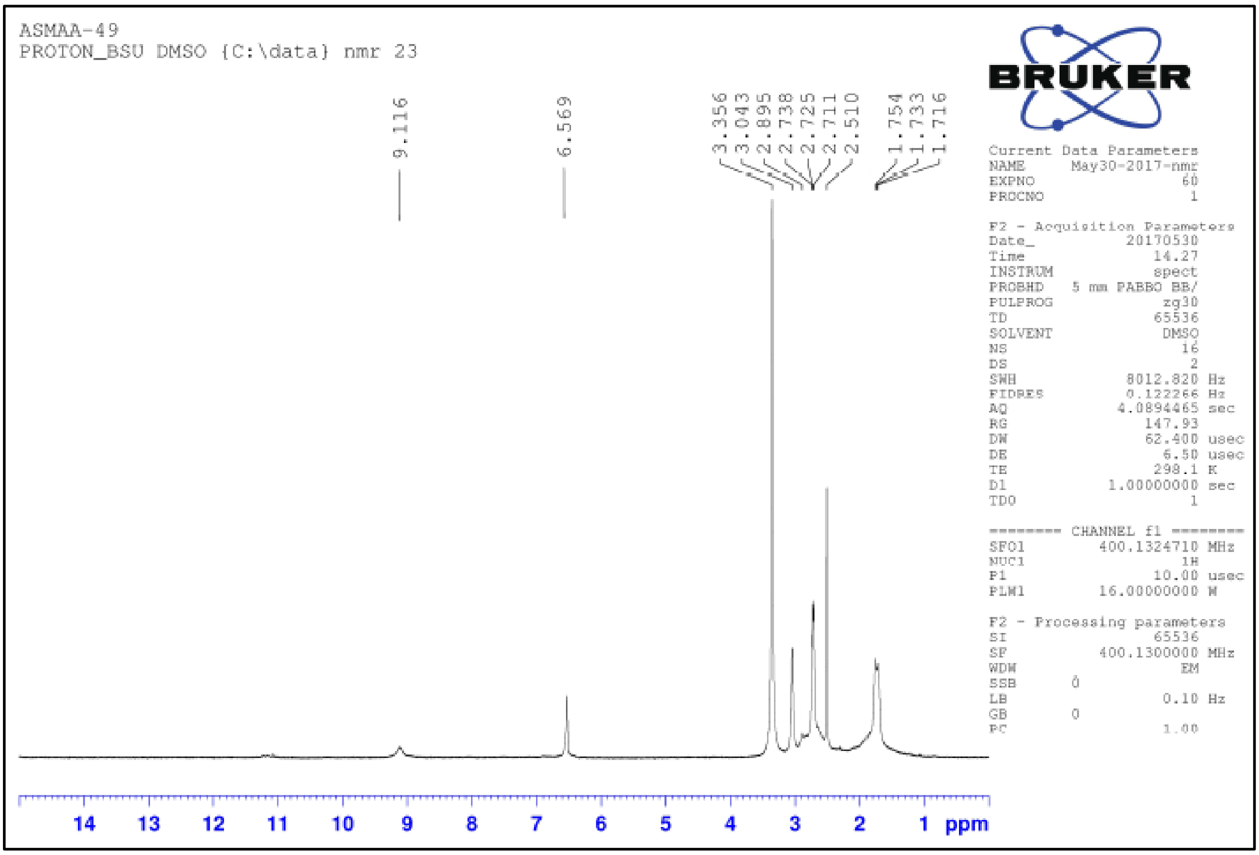

Fig. S8. ^{1}H NMR spectrum of compound **(6c)**

References

1. Khalifa, M. E.; Elkhawass, E. A.; Pardede, A.; Ninomiya, M.; Tanaka, K.; Koketsu, M., A facile synthesis of formazan dyes conjugated with plasmonic nanoparticles as photosensitizers in photodynamic therapy against leukemia cell line. *Monatsh. Chem.* **2018,** 149, 2195-2206.
2. Chen, Y.; Liew, K. Y.; Li, J., Size-controlled synthesis of Ru nanoparticles by ethylene glycol reduction. *Mater. Lett.* **2008,** 62, 1018-1021.
3. Kusada, K.; Kobayashi, H.; Yamamoto, T.; Matsumura, S.; Sumi, N.; Sato, K.; Nagaoka, K.; Kubota, Y.; Kitagawa, H., Discovery of Face-Centered-Cubic Ruthenium Nanoparticles: Facile Size-Controlled Synthesis Using the Chemical Reduction Method. *J. Am. Chem. Soc.* **2013,** 135, 5493-5496.
4. Frisch, M.; Trucks, G.; Schlegel, H.; Scuseria, G.; Robb, M.; Cheeseman, J.; Scalmani, G.; Barone, V.; Mennucci, B.; Petersson, G., Gaussian 09, Revision A. 1. *Wallingford, CT, USA: Gaussian.* **2009**.
5. Becke, A. D., Density-functional thermochemistry. III. The role of exact exchange. *J. Chem. Phys.* **1993,** 98, 5648-5652.
6. Lee, C.; Yang, W.; Parr, R. G., Development of the Colle-Salvetti correlation-energy formula into a functional of the electron density. *Phys. Rev. B* **1988,** 37, 785.
7. Perdew, J. P.; Wang, Y., Pair-distribution function and its coupling-constant average for the spin-polarized electron gas. *Phys. Rev. B* **1992,** 46, 12947.
8. Dennington, R.; Keith, T.; Millam, J., GaussView, version 5. *Semichem Inc., Shawnee Mission, KS* **2009**.
9. BIOVIA, D. S., Materials Studio 2017. *Dassault Systèmes, San Diego Google Scholar* **2017**.
10. Delley, B., Ground-state enthalpies: evaluation of electronic structure approaches with emphasis on the density functional method. *J. Phys. Chem. A* **2006,** 110, 13632-13639.
11. Perdew, J. P.; Burke, K.; Ernzerhof, M., Generalized Gradient Approximation Made Simple. *Physical Review Letters* **1996,** 77, 3865-3868.
12. Ito, S.; Chen, P.; Comte, P.; Nazeeruddin, M. K.; Liska, P.; Péchy, P.; Grätzel, M., Fabrication of screen-printing pastes from TiO2 powders for dye-sensitised solar cells. *Progress in Photovoltaics: Research and Applications* **2007,** 15, 603-612.
13. Kavan, L.; Krysova, H.; Janda, P.; Tarabkova, H.; Saygili, Y.; Freitag, M.; Zakeeruddin, S. M.; Hagfeldt, A.; Grätzel, M., Novel highly active Pt/graphene catalyst for cathodes of Cu (II/I)-mediated dye-sensitized solar cells. *Electrochim. Acta* **2017,** 251, 167-175.
14. Ferdowsi, P.; Saygili, Y.; Zakeeruddin, S. M.; Mokhtari, J.; Grätzel, M.; Hagfeldt, A.; Kavan, L., Alternative bases to 4-tert-butylpyridine for dye-sensitized solar cells employing copper redox mediator. *Electrochim. Acta* **2018,** 265, 194-201.
